# Molecular characterization of thioester-containing proteins in *Biomphalaria glabrata* and their differential gene expression upon *Schistosoma mansoni* exposure

**DOI:** 10.3389/fimmu.2022.903158

**Published:** 2022-07-27

**Authors:** J. Marquez, N. Dinguirard, A. Gonzalez, A.E. Kane, N.R. Joffe, T.P. Yoshino, M.G. Castillo

**Affiliations:** ^1^ Department of Biology, New Mexico State University, Las Cruces, NM, United States; ^2^ Department of Pathobiological Sciences, School of Veterinary Medicine, University of Wisconsin-Madison, Madison, WI, United States

**Keywords:** *Biomphalaria glabrata*, differential gene expression, innate immunity, resistance, *Schistosoma mansoni*, schistosomiasis, susceptibility, thioester-containing proteins

## Abstract

Schistosomiasis is a disease caused by trematode parasites of the genus *Schistosoma* that affects approximately 200 million people worldwide. Schistosomiasis has been a persistent problem in endemic areas as there is no vaccine available, currently used anti-helmintic medications do not prevent reinfection, and most concerning, drug resistance has been documented in laboratory and field isolates. Thus, alternative approaches to curtail this human disease are warranted. Understanding the immunobiology of the obligate intermediate host of these parasites, which include the freshwater snail *Biomphalaria glabrata*, may facilitate the development of novel methods to stop or reduce transmission to humans. Molecules from the thioester-containing protein (TEP) superfamily have been shown to be involved in immunological functions in many animals including corals and humans. In this study we identified, characterized, and compared TEP transcripts and their expression upon *S. mansoni* exposure in resistant and susceptible strains of *B. glabrata* snails. Results showed the expression of 11 unique TEPs in *B. glabrata* snails. These transcripts present high sequence identity at the nucleotide and putative amino acid levels between susceptible and resistant strains. Further analysis revealed differences in several TEPs’ constitutive expression levels between resistant and susceptible snail strains, with C3-1, C3-3, and CD109 having higher constitutive expression levels in the resistant (BS90) strain, whereas C3-2 and TEP-1 showed higher constitutive expression levels in the susceptible (NMRI) strain. Furthermore, TEP-specific response to *S. mansoni* miracidia exposure reiterated their differential expression, with resistant snails upregulating the expression of both TEP-4 and TEP-3 at 2 h and 48 h post-exposure, respectively. Further understanding the diverse TEP genes and their functions in invertebrate animal vectors will not only expand our knowledge in regard to this ancient family of immune proteins, but also offer the opportunity to identify novel molecular targets that could aid in the efforts to develop control methods to reduce schistosomiasis transmission.

## Introduction

### Schistosomiasis, *S*. *mansoni*, and *B*. *glabrata*

Schistosomiasis is a neglected tropical disease caused by trematode parasites of the genus *Schistosoma*. In 2018, the World Health Organization (1) estimated that approximately 229 million people globally are affected by this disease, with most infections in the Americas and Africa being due to *Schistosoma mansoni*. Schistosomiasis predominantly affects low-income rural populations, mostly in developing areas where adequate sanitation, access to clean water and/or medical infrastructure are lacking (2, 3). Currently there are no vaccines available, and although an effective drug, praziquantel, exists and is in wide usage, its inability to prevent reinfection when discontinued, and reports of drug resistance in both laboratory and field settings (4–6) raises concerns regarding the drug’s future efficacy. Therefore it is critically important that alternative approaches to controlling spread of schistosomiasis in vulnerable human populations be pursued.

Schistosome transmission is dependent on infection of an obligatory aquatic intermediate molluscan host, in which asexual reproduction to the human-infective cercaria stage takes place. Free-swimming cercariae are then released to infect humans in contact with contaminated water. For this study, we utilized the snail *Biomphalaria glabrata* as it is the predominant intermediate host for *S. mansoni* in the Americas and has been a useful laboratory model in the study of snail-schistosome interactions for several decades (7–10). A variety of *B. glabrata* strains have been shown to have different levels of susceptibility to *S. mansoni* infection, which has been shown to be, in part, genetically based (11). Therefore, studying the natural resistance of the intermediate snail host at the genetic and molecular levels is an approach that could lead to alternative measures to control human infections. Transcriptomic studies using *B. glabrata* tissues during immune challenge have identified differentially expressed genes that are important in stress and defense (12, 13) including reactive oxygen species (ROS) (14, 15), fibrinogen-related proteins (FREPs) (16–18), lectins (19–21), and thioester-containing proteins (TEPs) (22, 23). Furthermore, recent studies have investigated the relationship and possible mechanistic associations between these and other immune-related molecules which in addition to TEPs, incorporate FREPs and biomphalysin in a stepwise recognition, binding and attacking *S. mansoni* sporocysts in resistant snails (24).

### Thioester-containing proteins

The family of TEPs are considered pattern recognition receptors (PRRs), that are involved in innate immunity and known to have a protective role against invading pathogenic organisms (25). In general, TEPs have several protein domains in common, including the characteristic thioester region (TER), with the exception of complement component C5 and some insect TEPs (26, 27). The TER contains a highly conserved amino acid (AA) sequence: (N)-glycine-cysteine-glycine-glutamate-glutamine-(C) (GCGEQ). Studies across species customarily organize the TEP superfamily into three major groups of proteins that reflect minor differences in their structural and functional characteristics. These three major groups are the insect thioester-containing proteins (iTEPs, here called TEPs), the complement component C3 (C3)-like group, and the alpha-2-macroglobulin (A2M) group (25, 28, 29). The TEP group is further subdivided into two subgroups: the classical TEPs and CD109 (25, 30). Although the classical TEPs were first identified in *Drosophila melanogaster* and initially characterized in insects, they have now been found in many other organisms (31). Furthermore, these proteins have been shown to be involved in innate immune function by acting against parasites and bacteria (25, 30, 32, 33). Diversity of TEPs was extensively studied in both the fruit fly *D. melanogaster* and the mosquito *Anopheles gambiae*, in which 6 and 19 TEPS were identified, respectively (34–36). Several studies have further characterized TEPs immune functions, demonstrating the role of *A. gambiae* TEP1 (AgTep1) in recognition, opsonization, and phagocytosis of microorganisms (25, 37). Interestingly, studies in larval and adult *D. melanogaster* revealed that TEPs are differentially expressed in tissues (38), suggesting functional specificity for these proteins. Finally, CD109 molecules are cell surface glycoproteins that can be found on immune cells such as hematopoietic stem cells, T-cells, endothelial cells, and platelets (28, 39), suggesting that CD109 molecules are also involved in immunity.

The second major group of the TEP superfamily are the C3-like proteins. These molecules have been well studied and characterized in vertebrates as well as several ancestral species including tunicates, ascidians, cnidarians, echinoderms, and arthropods (32, 40–42). Complement component C3 acts as the central molecule that merges the three functional complement pathways in vertebrates (43–45). There are many reported immunological functions resulting from the activation of the complement cascade, including inflammation, chemoattraction, cytokine secretion, opsonization, phagocytosis, lymphocyte activation, and cell lysis. (44–46).

The third major division in the TEP superfamily is the alpha-2-macroglobulin (A2M) group, which is composed of three related types of proteins: the pan-protease inhibitor A2M, CPAMD8 (C3 and PZP-like alpha-2-macroglobulin domain-containing protein 8), and PZP (pregnancy-zone protein) molecules. A2Ms are thought to be involved in some of the most evolutionary ancient defense mechanisms; they have been identified in prokaryotes and with similar function to that of vertebrate and invertebrate A2M/C3b homologs (47). A2Ms are protease inhibitors that act as opsonins (48, 49) and were more recently described as having chaperone functions (50). CPAMD8 is a cell-bound protein and combines structural characteristics of complement and A2M proteins, resulting in a molecule with functional serine-protease and thioester domains for protease control and induction of inflammation (51). Finally, PZP was initially considered a protease inhibitor found in plasma of healthy individuals but with a significant higher concentration in pregnant women (52), was also recently described as a chaperone that aids in the removal of pro-inflammatory proteins (50).

Like other PRRs, TEP responses depend on the recognition of structural motifs that are conserved in pathogen-associated molecular patterns (PAMPs). Studies analyzing and characterizing the tick and fruit fly immune response to different pathogens showed a TEP-specific response to each pathogen, suggesting that different members of the TEP superfamily were discerning between the various PAMPs presented and responding accordingly (33, 53–55).

### TEPs in *B. glabrata*

In *B. glabrata*, the first TEP molecule was identified in 2008 (GenBank: FJ480411.1), and later characterized in association with the recognition and response to surface *S. mansoni* epitopes (22, 23). This molecule, designated as TEP-1 for the remainder of this study, is grouped with other TEP/CD109 homologues based on phylogenetic analysis. Two recent publications describe the diversity of TEPs in *B. glabrata*, one from Duval etal. (56) using the Bre1 strain, and a review by our group in the susceptible BB02 strain (10). These studies support the idea that *B. glabrata* strains have a complex array of immune mechanisms that include a diverse group of molecules within the TEP superfamily.

The goal of this study was to verify the transcripts of the 11 TEPs initially identified in the *B. glabrata* genomic database, compare their sequences, and characterize the differences between susceptible and resistant snail strains. This study also aimed to assess how these TEPs are differentially expressed in snails exposed to *S. mansoni* miracidia. Results from this study will further our understanding of TEPs functionality in *B. glabrata* snails and may serve as a step towards elucidating the molecular basis of the schistosome-susceptibility/resistance differences observed among snail strains.

## Material and methods

### Ethics statement

Mice used in this study were maintained in animal housing facilities at Charmany Instructional Facility (University of Wisconsin-Madison) approved by the American Association for Accreditation of Laboratory Animal Care (AAALAC) and using standard-of-care cage housing and feeding. All animal handling and experimental protocols were performed under approval by the University of Wisconsin-Madison Institutional Animal Care and Use Committee (IACUC, Animal Welfare Assurance No. A3368-01).

### Live materials

*B. glabrata* snails, resistant (BS90) and susceptible (BB02 and NMRI) strains, were reared and maintained separately in the laboratories at New Mexico State University and the University of Wisconsin-Madison. Snails were kept in 10-gallon aquaria with aerated artificial pond water (APW: 124.89 mM CaCO_3_, 14.83 mM MgCO_3_, 21.39 mM NaCl, and 3.35 mM KCl) at approximately 26°C, under a 12-h light-dark cycle, and fed a diet of romaine lettuce three times a week, supplemented with fish flakes twice a week, and edible chalk (as needed).

### Isolation of miracidia and snails exposure

Mice, infected with the NMRI strain of *S. mansoni*, were provided by the Biomedical Research Institute (Rockville, MD). At 7 weeks post-infection, mice were euthanized and their livers removed for axenic egg isolation following protocols previously published (57, 58). Briefly, dissected livers were blended using phosphate buffered saline (PBS) with antibiotics. The lysate was then transferred to a Florence flask covered with aluminum foil, except for the top two inches, where a light source was shined to collect swimming miracidia from the surface of the liquid. Miracidia were counted by taking three 10 uL-aliquots and viewed under a microscope. Then the average count was used to calculate the infective dose to expose snails.

A total of 108 *B. glabrata* snails were used in exposure experiments, 54 from the NMRI strain (susceptible) and 54 from the BS90 strain (resistant). Each exposure experiment consisted of 18 snails per strain, with 9 snails exposed to miracidia and 9 snails kept in APW only. Snails were exposed to miracidia for 1 h and after 2, 12 and 48 h from original exposure, three snails per treatment and strain, were randomly selected and sacrificed for RNA extraction.

After one h, the wells of exposed snails were checked by microscopic observation to verify miracidial penetration. After which all snails were moved to four different 1L beakers containing pond water (NMRI exposed, NMRI control, BS90 exposed, BS90 control) until sacrificed at their corresponding time points. Three snails were randomly selected from each treatment group and sacrificed at 2, 12, and 48 h post-miracidial exposure. Each exposure experiment was repeated three times.

### RNA extraction, DNase treatment, and cDNA synthesis

For sequencing purposes, a minimum of three snails of 10-14 mm in diameter from both BB02 and BS90 strains were sacrificed by removing the shell and individually placed into 1.5 mL microcentrifuge tubes containing 0.5 mL Trizol reagent (Life Technologies) and homogenized using a plastic pestle and electric homogenizer (Kimble). Total RNA was extracted following the manufacturer’s instructions, with the exception of the isopropanol incubation extended overnight at -20°C, and the ethanol wash step performed twice. Total RNA concentrations were measured using a Nanodrop 1000 spectrophotometer (Thermo Fisher Scientific). Total RNA samples were then subjected to DNase treatment using the Turbo DNase-free Kit (Life Technologies). Concentration and quality of the resulting DNA-free RNA samples were assessed using the Eppendorf BioPhotometer plus, and then stored at -80°C until needed. DNA-free RNA samples were subjected to cDNA synthesis using the First-Strand cDNA Synthesis kit (Promega).

### Polymerase chain reaction and rapid amplification of cDNA ends

TEP sequences identified in the BB02 reference genome were used as template to design PCR, qRT-PCR, and 5’- and 3’-RACE primers with the National Center for Biotechnology Information’s (NCBI) Primer-BLAST and Integrated DNA Technologies (IDT) PrimerQuest online tools. Primers were purchased from the Custom DNA Oligo Synthesis service (Eurofins Genomics) (**Supplemental Table 1**). cDNA samples from each snail strain were then used to amplify each TEP using PCR and GoTaq G2 Green Master Mix (Promega). In addition, the Q5 High-Fidelity 2X Master Mix (New England Biolabs) was also used to verify the fidelity of the original sequences using several PCR primer sets (**Supplemental Tables 1**–**3**). Primers designed for RACE (**Supplemental Table 4**) were used to extend the unknown 5’ and 3’ ends of incomplete sequences using the GeneRacer kit (Thermo Fisher Scientific). Transcripts of interest included 11 members of the TEP superfamily identified using the BB02 reference genome (A2M-1, A2M-2, CPAMD8, C3-1, C3-2, C3-3, TEP-1, TEP-2, TEP-3, TEP-4, and CD109) as reported in Castillo et al. (10). As a positive control, the housekeeping gene, ribosomal protein subunit 19 (RPS19) was also amplified.

### Agarose gel electrophoresis, PCR purification, and sequencing

PCR, qRT-PCR and RACE products were analyzed with gel electrophoresis (1.2% agarose (OmniPur, EMD) and the GeneRuler-Low Range DNA Ladder (Thermo Fisher Scientific) for size reference. Upon separation, the product bands were visualized using a UV-light imaging unit (UVP GelStudio PLUS, Analytik Jena) and saved electronically. Amplified products using qRT-PCR primer pairs were also analyzed similarly to ensure primers amplified and matched expected product size before proceeding to qRT-PCR protocols. PCR and RACE products matching expected sizes were purified using the PureLink PCR Purification Kit (Life Technologies) and submitted for Sanger Sequencing using the SimpleSeq DNA Sequencing Service (Eurofins Genomics).

### Sequence assemblage, characterization, and phylogenetic analysis

Forward and reverse sequencing files were assembled into contigs and their corresponding consensus obtained using several bioinformatics tools including the Sequence Manipulation Suite (https://www.bioinformatics.org/sms2/) and Vector NTI Advance software (Thermo Fisher Scientific). Sequences from the *B. glabrata* BB02 reference genome in VectorBase (9) were used as template guides. A translation tool (ExPASy – Bioinformatics Resource Portal) was used to obtain putative amino acid (AA) sequences. NCBI Protein BLAST tool was used to obtain the closest TEP homologs, as well as to identify TEP functional domains using the conserved domain database (CDD) (Lu S. et al., 2020; http://www.ncbi.nlm.nih.gov/cdd), and to identify the AA mismatches between BB02 and BS90 sequences. Conserved domains were confirmed with SMART tool (http://smart.embl-heidelberg.de/), while SignalP 4.0 Server (http://www.cbs.dtu.dk/services/SignalP-4.0/) was used for the identification of signal peptide sequences, and the GPI-SOM Server (http://gpi.unibe.ch/) for the identification of glycosylphosphatidylinositol (GPI)-anchors. Lastly, all TEP sequences were aligned with Multiple Sequence Comparison by Log-Expectation (MUSCLE) to show a graphic comparison of the TEP TER motif between BB02 and BS90 sequences. Note that all of the nucleotide (NT) and protein (AA) sequence alignments and data analysis were performed using coding sequences from start to stop codons if the complete sequence was obtained, or the longest coding sequence available if it was incomplete.

**Figure 1:**
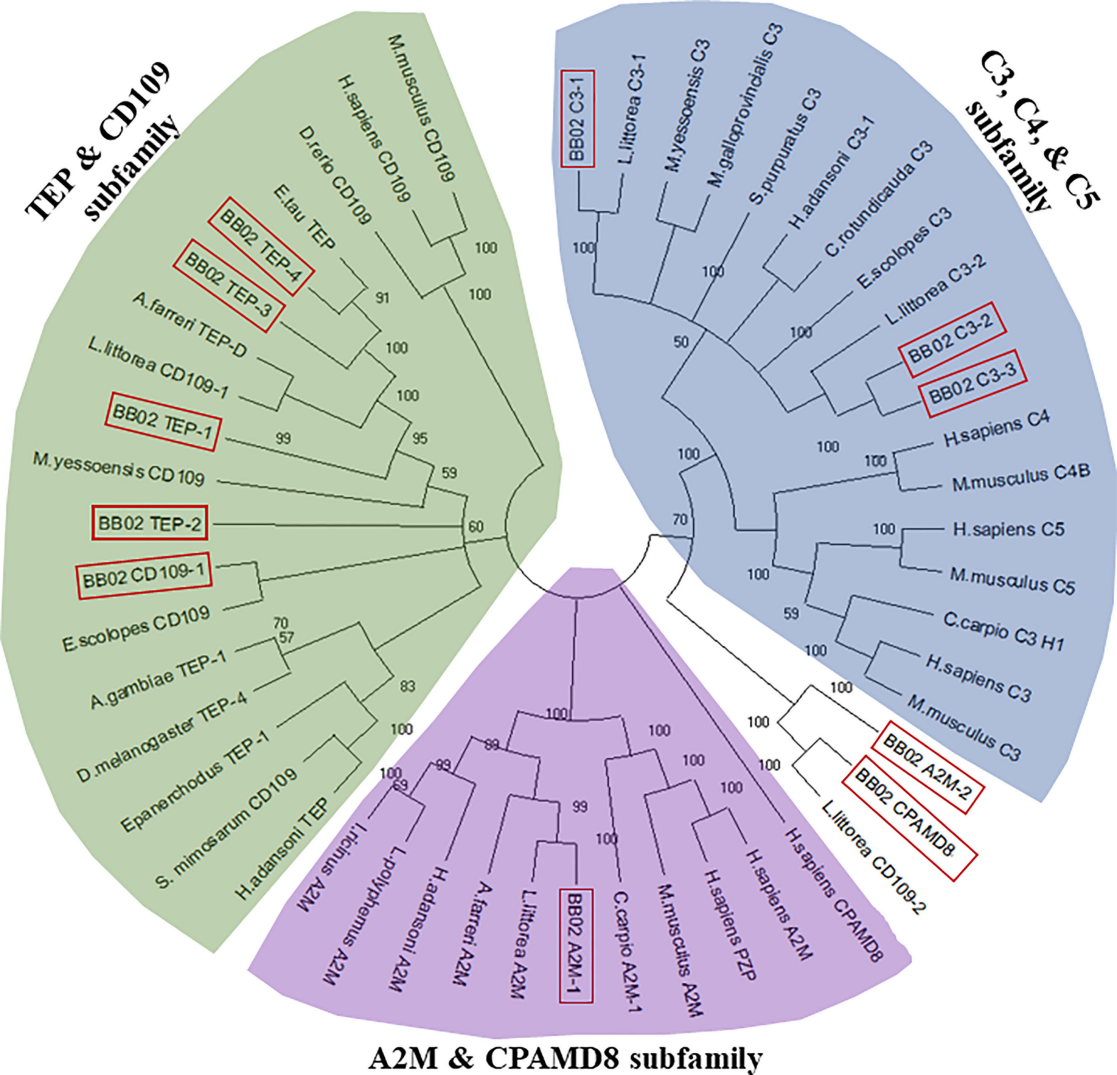
Phylogenetic tree of the *B. glabrata* TEPs among other homologs in the TEP superfamily. BB02 and BS90 *B. glabrata* TEP protein sequences were aligned with MUSCLE and a phylogenetic tree was constructed utilizing 39 additional full-length TEP protein homologs from diverse animal species. The neighbor-joining statistical method (60), James-Thornton-Taylor model (61), and bootstrap phylogeny test with 500 replications were utilized to construct the unrooted tree with MEGA-X (59). *B. glabrata* BB02 protein sequences are marked in red boxes. The homolog sequences used and their accession numbers can be found in **Supplemental Table 5**.

Assembled *B. glabrata* TEP AA sequences were compared and aligned with MUSCLE against known TEP homologs from various vertebrate and invertebrate organisms (**Supplemental Table 5**) to determine sequence relationships and to construct an unrooted phylogenetic tree using the Molecular Evolutionary Genetics Analysis (Mega)-X software, version 10.0.5 (59). The Neighbor-Joining tree model (60), James-Thornton-Taylor model (61) was constructed using a Bootstrap value of 500 replications (**Figure 1**). An additional phylogenetic tree (**Supplemental Figure 2**) was constructed to compare the BB02 *B. glabrata* TEP sequences to those reported from the Bre1 strain (23, 56).

### Quantitative real-time PCR and analysis

The head foot region of 108 individual snails (three snails per time point/treatment/strain) from the three exposure experiments previously described (section 2.3) were dissected and individually used for total RNA isolation and subsequent DNase treatment as described above in section 2.4. DNase-treated RNA was normalized to the sample with the lowest concentration during the cDNA preparation, and then diluted to a final concentration of 5 ng/µL. The constituents in each qPCR reaction included 10 µL of Sybr Green PCR Master Mix (Applied Biosystems), 4 µL of each optimized forward and reverse primers, and 10 ng of cDNA. All samples were run with two technical replicates in 96-well Optical Reaction Plates (ABI PRISM, Applied Biosystems). The reaction plates were loaded into AB7500 Real time PCR System (Applied Biosystems). All qRT-PCR reactions were run at an annealing temperature of 55°C using primers at a concentration of 350 nM, except the RPS19 control gene (150 nM) (**Supplemental Table 6**). TEPs gene expression was normalized to the reference gene, RPS19 (ΔCT = CT_target_ – CT_reference_). To compare the differences in constitutive TEPs gene expression between BS90 and NMRI snail strains, log-transformed expression levels of BS90 control snails were calculated relative to NMRI control snails using the Pfaffl comparative method across all time points (62). To compare TEPs gene expression between BS90 and NMRI snail strains post-exposure at 2, 12, and 48 h, log-transformed expression levels of exposed snails were calculated relative to control snails for each strain using the Pfaffl comparative method. Statistical analysis for both comparisons were performed using the non-parametric Mann-Whitney test due to non-normal distribution of the data. Values were considered significant if p ≤ 0.05.

## Results

### *B. glabrata* nucleotide and protein sequence characterization

Using the *B. glabrata* genome and its associated datasets (9), along with the results obtained from PCR and RACE, we have identified and assembled 11 unique members of the TEP superfamily in both the BB02 and BS90 snail strains. These include three members from the A2M group (A2M-1, A2M-2, CPAMD8); three complement C3-like molecules (C3-1, C3-2, C3-3), and five classical TEP-related proteins (TEP-1, TEP-2,TEP-3, TEP-4, and CD109). **Table 1** includes the list of these sequences along with their corresponding GenBank accession numbers, with four of these sequences (A2M-2, CPAMD8, C3-3, and TEP-3) only partially verified. The corresponding BGLB ID (nucleotide), NCBI predicted sequence match (protein), and the closest homolog (protein) for each *B. glabrata* TEP were chosen based on the highest cover query and percent identities after local blast analysis. The *B. glabrata* predicted sequences found in the NCBI database did not always match with the given name of the closest homolog from other species, although all remained within the same TEP superfamily subgroup (**Table 1**).

**Table 1:** *B. glabrata* BB02 and BS90 sequence identifiers.

TEP	BB02 Accession	BS90 Accession	Complete	BGLB ID	NCBI Predicted	Closest Homolog
A2M-1	MK573558	MW557847	Yes	016521-RB	XP_013081252.1 (96%) alpha-1-inhibitor 3-like	A2M *L. littorea* AIC31934.1 (46%)
A2M-2	MK576002	MW752363	No	3’ end – 022655-RA	XP_013084477.1 (100%) alpha-2-macroglobulin-like	CD109-2 *L. littorea* AVP12647.1 (55%)
CPAMD8	MK576004	MW752364	No	3’ end – 035268-RA	XP_013061675.1 (100%) CPAMD8	CD109-2 *L. littorea* AVP12647.1 (62%)
C3-1	MK583200	MW752365	Yes	018444-RA	XP_013068508.1 (100%) complement C3-like	C3 *L. littorea* AVP12644.1 (54%)
C3-2	MK583201	MW752366	Yes	5’ end – 030610-RA 3’ end – 020436-RA	XP_013086914.1 (97%) venom factor 1-like	C3 *L. littorea* AVP12645.1 (33%)
C3-3	MK583202	MW752367	No	5’ end – 021062-RA 3’ end – 025256-RA	XP_013064315.1 (100%) complement C5-like	C3 *L. littorea* AVP12645.1 (36%)
TEP-1	MK583203	MW752368	Yes	5’ end – 021162-RA 3’ end – 035158-RA	ADE45332.1 (97%) thioester-containing protein	CD109 *L. littorea* AVP12646.1 (38%)
TEP-2	MK583204	MW752369	Yes	5’ end – 000155-RA 3’ end – 032760-RA	XP_013065920.1 (93%) CD109 antigen-like	CD109 *M. yessoensis* OWF38485.1 (32%)
TEP-3	MK583205	MW752370	No	5’ end – 000023-RB	XP_013091771.1 (99%) CD109 antigen-like	TEP *E. tau* BAE44110.1 (53%)
TEP-4	MK583206	MW752371	Yes	5' end – 030043-RB 3' end – 021854-RA	XP_013071291.1 (100%) CD109 antigen-like	TEP *E. tau* BAE44110.1 (69%)
CD109	MK576003	MW752372	Yes	5’ end – 021085-RA 3’ end – 031746-RA	XP_013094127.1 (98%) CD109 antigen-like	CD109 *S. mimosarum* KFM64970.1 (29%)

BGLB ID 5' and 3' are listed only if each sequence end had a different BGLB ID. BB02 and BS90 snail strains contained the same sequence identifiers, except the GenBank accession numbers.

To further characterize the relationship between these 11 *B. glabrata* sequences and other members of the TEP superfamily, a phylogenetic analysis was conducted using the *B. glabrata* TEP amino acid (AA) sequences with those of their corresponding closest homologs and other known vertebrate and invertebrate sequences within the TEP superfamily (**Figure 1**). Sequences from the BS90 strain were not included in this analysis for simplicity, as both BB02 and BS90 TEPs were found to have high identities at the nucleotide (NT) and protein levels. The phylogenetic tree showed that 9 out of the 11 *B. glabrata* TEP protein sequences grouped into the three traditional A2M, C3, and TEP subfamily clades (**Figure 1**). The remaining two TEPs (A2M-2 and CPAMD8) were placed in an undefined group, most possibly because these sequences are incomplete, and thus, alignment and phylogenetic analysis are not optimal. For these two sequences, the best NCBI-BLAST match was used as the identifier. The characterization of *B. glabrata* TEPs presented in this study is purely based on the identification of specific sequence domains and motifs, as functional studies are needed to fully characterize them.

### BB02 and BS90 TEP nucleotide and amino acid sequence comparisons

#### Sequence length

Those *B. glabrata* TEPs for which we obtained complete coding sequences, the NT and protein sequences ranged from 4164-5265 base pairs and 1387-1754 AAs, respectively (**Table 2**). The NT lengths listed include stop codons, except for the four incomplete sequences (A2M-2, C3-3, CPAMD8, and TEP-3). The only sequences that showed a difference in length between the BB02 and BS90 strains were TEP-1 and TEP-3. At the protein level, these differences translated to three additional AA in BB02 TEP-1 compared to BS90 (**Table 2\**, discussed below), whereas BS90 TEP-3 contained one extra AA compared to the BB02 sequence. To verify these differences, PCR was conducted with several biological replicates of both snail strains using the Q5 high-fidelity enzyme. Results confirmed the difference within these sites.

**Table 2:** BB02 and BS90 sequence lengths and identities.

TEP	BB02 Nucleotide Sequence Length	BS90 Nucleotide Sequence Length	Nucleotide Sequence Identity between BB02 and BS90 (Mismatches #)	BB02 Amino Acid Sequence Length	BS90 Amino Acid Sequence Length	Amino Acid Sequence identity between BB02 and BS90 (Mismatches #)
A2M-1	4860	4860	99.4% (27)	1619	1619	99.4% (9)
A2M-2	1968	1968	100% (0)	655	655	100% (0)
CPAMD8	1644	1644	99.7% (5)	548	548	99.6% (2)
C3-1	5175	5175	98.6% (70)	1724	1724	99.7% (6)
C3-2	5265	5265	99.5% (27)	1754	1754	99.4% (11)
C3-3	3849	3849	99.8% (6)	1283	1283	99.8% (3)
*TEP-1	4338	4329	98.3% (75)	1445	1442	96.7% (47)
TEP-2	4560	4560	98.7% (61)	1519	1519	97% (45)
*TEP-3	3564	3567	97.8% (78)	1188	1189	96.5% (42)
TEP-4	4164	4164	98.9% (44)	1387	1387	99.4% (9)
CD109	4365	4365	99.9% (6)	1454	1454	99.9% (2)

* Represents BB02 and BS90 different sequence lengths. nt, nucleotide; aa, amino acid.

#### Percent identity

*B. glabrata* TEPs’ NT and AA sequences were aligned using NCBI’s local blastn and blastp, respectively, and the global alignment Needleman-Wunsch tool. Results from the alignments revealed a 97.8-100% identity range from across their sequence lengths at the NT level, and between 96.7-100% identity at the AA level (**Table 2**). The most notable strain differences when comparing BB02 and BS90 TEP sequences were: 1) C3-1 and TEP-4, contained many NT differences (70 and 44, respectively), which appear to be synonymous replacements as only a few AA changed (6 and 9 respectively); and 2) TEPs-1, -2, and -3, had 75, 61, and 78 NT differences respectively, with over 50% of these corresponding to AA differences (47, 45, and 42, respectively).

#### Protein domains

**Figure 2:**
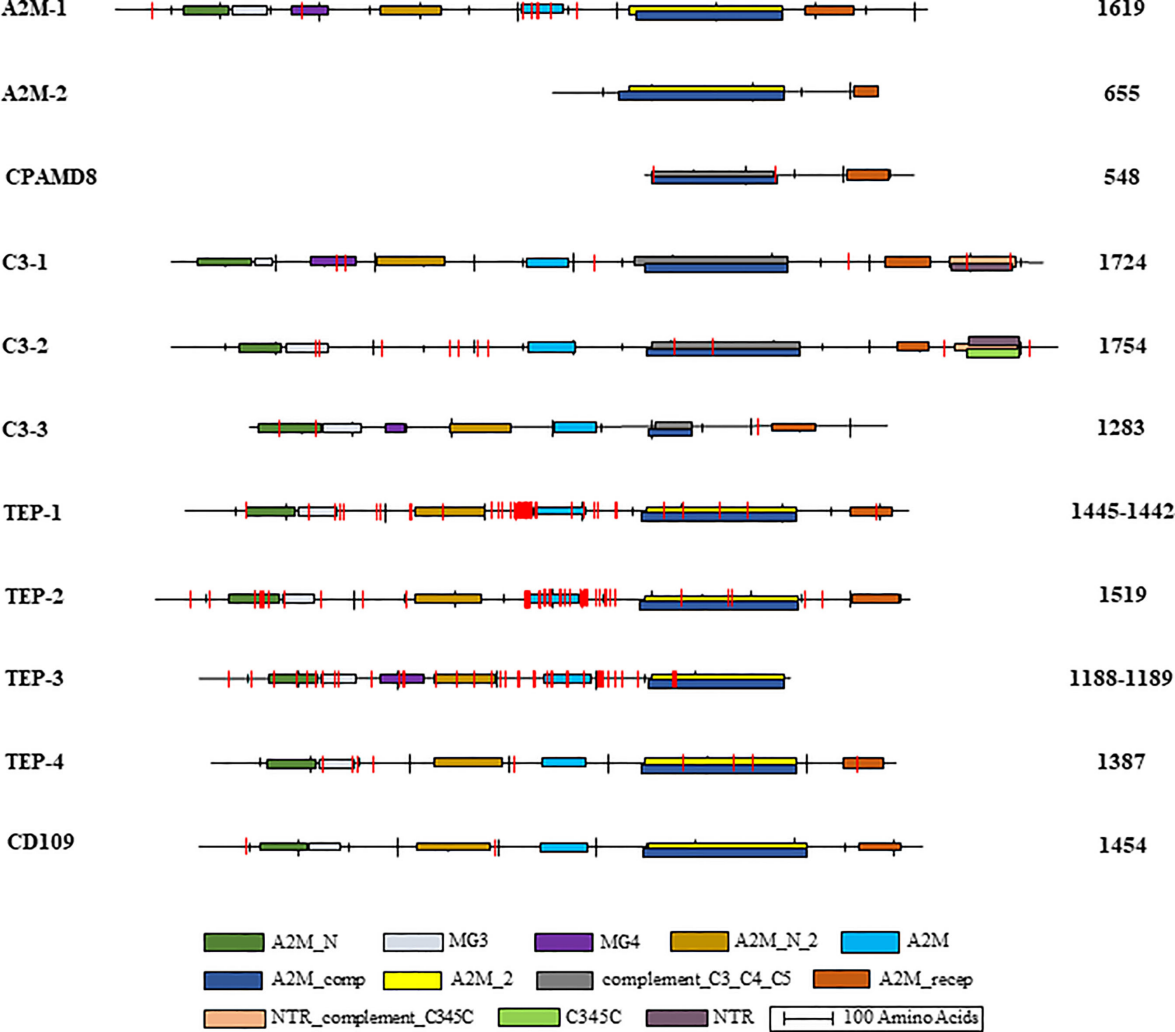
*B. glabrata* TEP structural domains and single amino acid mismatches. Graphical representation of the identified conserved domains in aligned *B. glabrata* BB02 and BS90 TEP amino acid sequences. The National Center for Biotechnology Information (NCBI) Conserved Domain Database **(CDD)** (63; http://www.ncbi.nlm.nih.gov/cdd) was utilized to localize TEP domains following their nomenclature and abbreviations: **A2M_N** = MG2 (macroglobulin) domain of alpha-2-macroglobulin; **MG3** = macroglobulin MG3 domain, corresponds to the MG3 domain found in complement components C3, C4 and C5; **MG4** = macroglobulin MG4 domain, found in complement C3 and C5 proteins; **A2M_N_2** = Alpha-2-macroglobulin family N-terminal region; **A2M** = Alpha-2-macroglobulin family, includes the C-terminal region of the alpha-2-macroglobulin family; **A2M_comp** = Complement component region of the alpha-2-macroglobulin family; **A2M_2** = Proteins similar to alpha-2-macroglobulin (alpha (2)-M). This group also contains the pregnancy zone protein (PZP), thioester region located within the structure; **complement_C3_C4_C5** = Proteins similar to C3, C4, and C5 of vertebrate complement, thioester region located within the structure of C3 and C4; **A2M_recep** = Receptor domain region of the alpha-2-macroglobulin family; **NTR_ complement_C345C** = NTR/C345C domain, NTR domains found in the C-termini of complement C3, C4, and C5; **C345C** = Netrin C-terminal Domain; **NTR** = UNC-6/NTR/C345C module, sequence similarity between netrin UNC-6 and C345C complement protein family members. The intervals of each domain and the length of each sequence are illustrated to scale. BB02 and BS90 TEP-1 and TEP-3 had different sequence lengths, so both are listed with BB02 first and BS90 second. The vertical red lines represent the relative location of amino acid mismatches.

Overall, BB02 and BS90 TEP sequences contained the same conserved protein domains and characteristic organization found in other vertebrate and invertebrate TEPs (**Figure 2**), albeit some sequences remain to be completed. It is to note that the NCBI’s CDD algorithm did not identify several domains in the *B. glabrata* complete TEP sequences. Based on domain architecture of similar proteins, A2M-1 contained all known domains, while in C3-2 the MG4 and A2M_N_2 domains were not identified. It is suspected that both the BB02 and BS90 C3-2 protein sequences have AAs in these areas that do not match other homologs as these regions tend to be highly variable, thus not allowing for their identification with the NCBI CDD algorithm. Similarly, in TEPs-1, -2, -4, and CD109 the MG4 domain was missing, but it was identified in TEP-3 (**Figure 2**).

#### Amino acid differences between strains

Amino acid (AA) mismatches among TEP sequences between the BB02 and BS90 snail strains were assessed using NCBI’s conserved domain database (CDD) at default settings, except using 20 domain homologs instead of 10 (63). AA mismatches are briefly discussed and are visually represented in **Figure 2**. A2M-1 and TEPs 1-3 are the molecules with higher number of mismatches between strains. The AA mismatches concentrated mainly in the bait region of A2M and the hypervariable region of TEPs (**Figure 2**). For example, TEP-1 sequences between BB02 and BS90 contained seven short gaps and 47 AA mismatches in total. Only 11 of those mismatches were in the A2M-conserved domain, and within a highly variable region (data not shown). The areas in TEP-1 containing gaps and mismatches corresponded to a region of low sequence similarity among these sequences as well as homologs from other species used in alignments. Interestingly, there were more AA mismatches when comparing BB02 and BS90 TEP-1 sequences than when comparing homologs of different species. Similarly, with TEP-2 and TEP-3, although the AA differences between snail strains sequences were highly concentrated in and around the A2M domain. Additionally, compared to TEPs 1-3, TEP-4 was more conserved between the two strains and did not have mismatches in the A2M domain, and only a few overall (data not shown).

In summary, all 11 domains characteristic of TEPs were identified in *B. glabrata* homologs with some mismatches between the two snail strains, but no clear pattern could be identified. However, most of the AA mismatches within conserved domains were also found to be divergent among homolog sequences in other organisms. It will be important to further assess the functional effect that these mismatches may have between snail strains. In addition, some of the differences between BB02 and BS90 involved substitutions containing AA residues with similar- R groups, and thus, folding of polypeptide chains may not be altered.

### Thioester region

**Figure 3:**
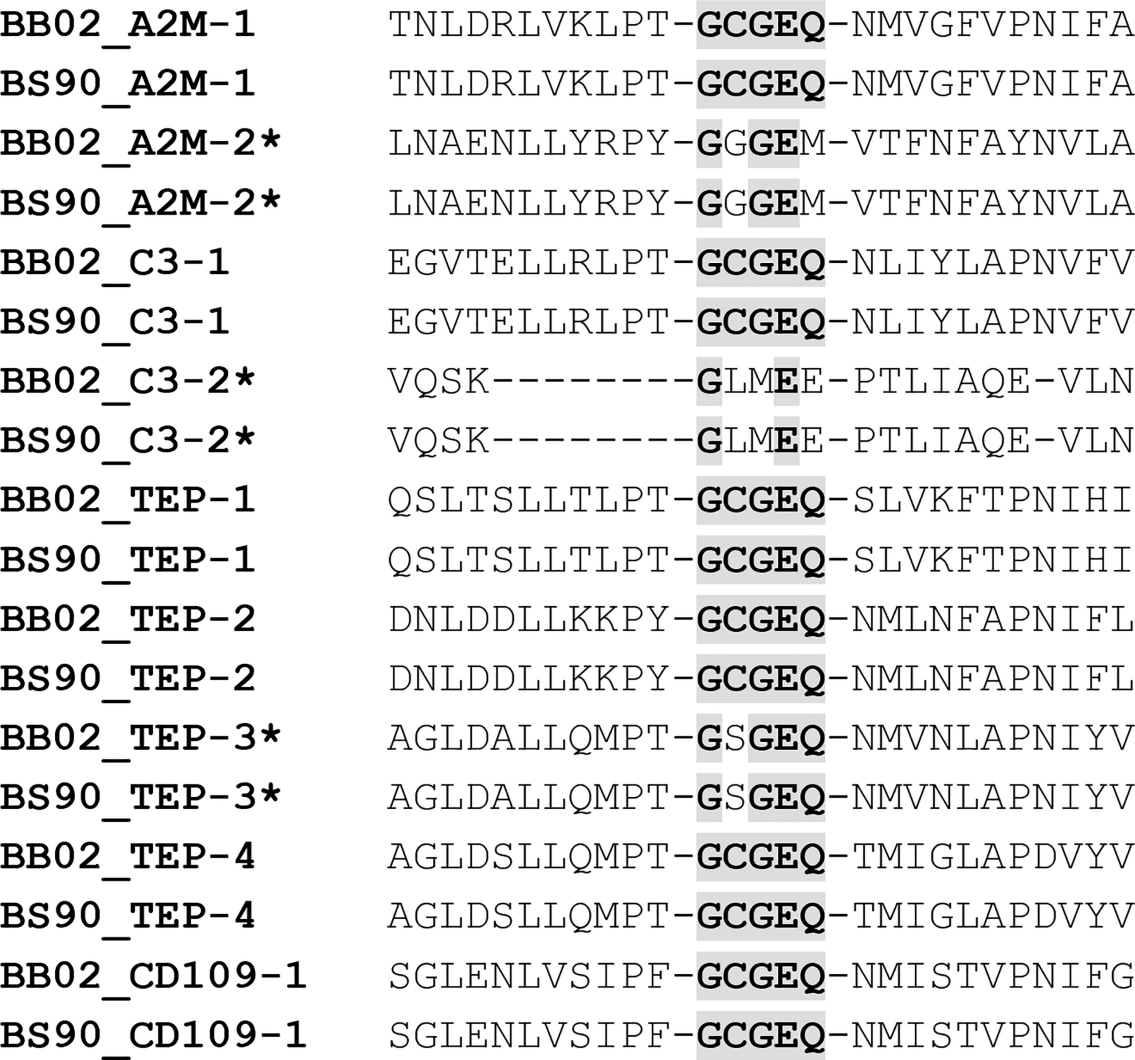
Presence of the Thioester Region (TER) in TEP Sequences. Amino acid alignment of BB02 and BS90 TEPs containing the characteristic GCGEQ TER. Conserved amino acids in the TER are highlighted and bolded, while TEPs containing one or more dissimilar amino acids within the TER are labeled with an asterisk. CPAMD8 and C3-3 were not included.

The TEP superfamily of proteins is characterized by several domains. Among these, the most important for the TEP classical function is carried out by the five-AA motif (G)CGEQ, also known as the thioester region (TER). This TER is usually contained near two overlapping domains: A2M_2 and complement_C3_C4_C5. NCBI’s CDD tool was used to identify the TER in BB02 and BS90 protein sequences, and then all sequences containing this motif were aligned with MUSCLE (**Figure 3**). Six out of the eleven TEPs in *B. glabrata* (both strains) contained the representative (G)CGEQ AA sequence. These included A2M-1, C3-1, TEP-1, TEP-2, TEP-4, and CD109. This suggests that these TEPs could function by binding foreign targets through a thioester bond. Interestingly, CPAMD8 and C3-3 had no identifiable TER, even though they both contained the complement_C3_C4_C5 domain, where the TER is typically located. It is possible that the TER in CPAMD8 is within the missing fragments of that sequence. Analysis of C3-3 showed that the complement_C3_C4_C5 interval was truncated, suggesting that a portion of this domain was lost (**Figure 2**). As a consequence of C3-3’s TER absence, the NCBI’s algorithm predicted it as a C5 homolog (**Table 1**). Furthermore, A2M-2, C3-2, and TEP-3 had alternative TER-amino acids: GGGEM, GLMEE, and GSGEQ respectively (**Figure 3**).

### Comparative TEPs expression between strains upon *S. mansoni* exposure

**Figure 4:**
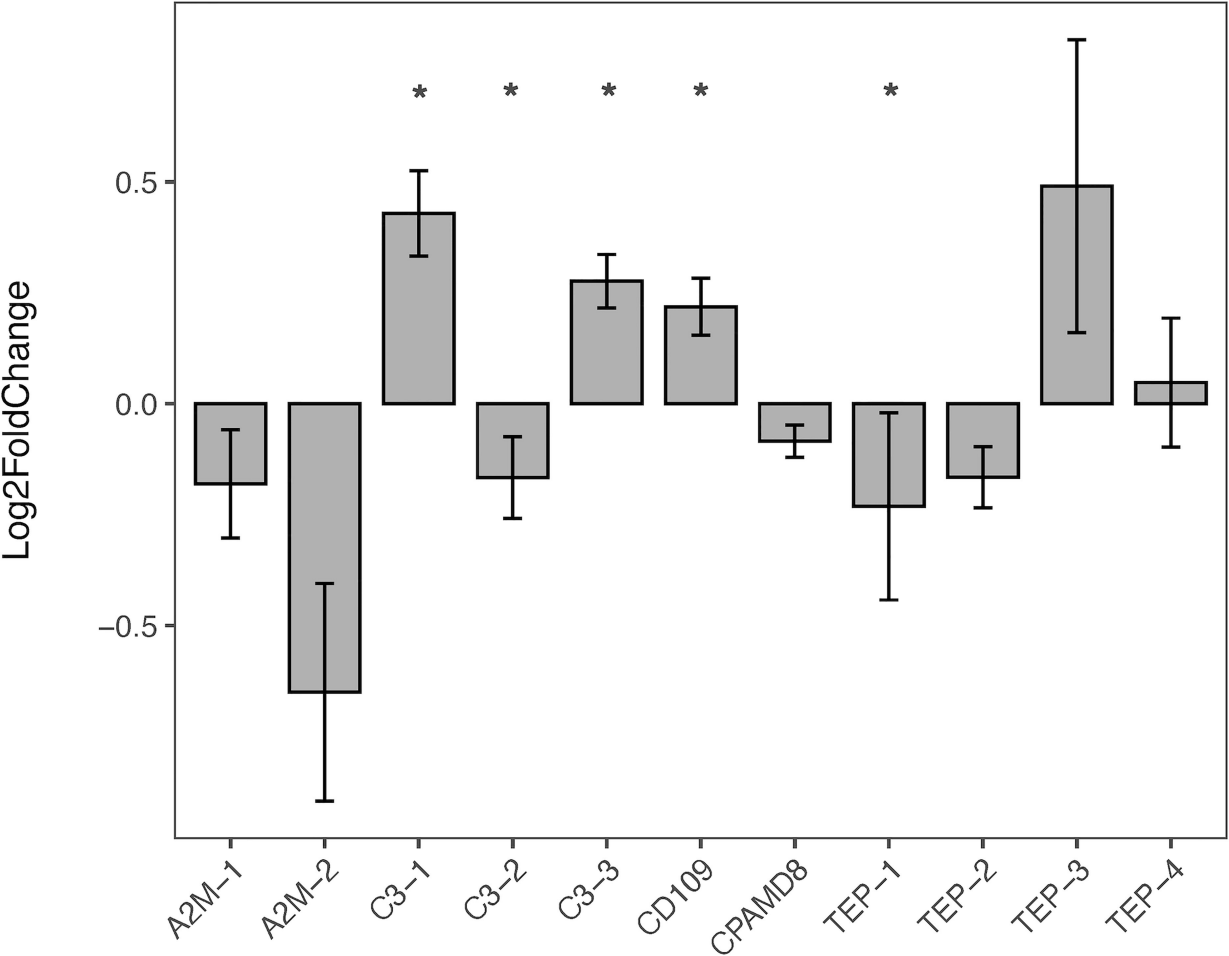
Constitutive Expression of TEPs in *B. glabrata*. The expression of the various TEP transcripts was measured *via* qRT-PCR in resistant BS90 snails (gray bars) and compared to susceptible NMRI snails (baseline) under control (no treatment) conditions. TEPs gene expression was normalized to the reference gene, RPS19 (ΔCT = CT_target_ – CT_reference_). To compare the differences in constitutive TEPs gene expression between BS90 and NMRI snail strains, log-transformed expression levels of BS90 control snails were calculated relative to NMRI control snails using the Pfaffl comparative method (across all time points; n=27). Statistical analysis was performed using the non-parametric Mann-Whitney test due to non-normal distribution of the data.* Indicates significant difference (p ≤ 0.05).

The expression of *B. glabrata* TEPs in response to *S. mansoni* miracidia was tested in resistant (BS90) and susceptible (NMRI) snails. In this study, TEPs’ modulation was examined after 2, 12, and 48 h post-exposure. Results showed that several TEPs are differentially expressed among these two strains. When analyzing the constitutive expression of TEPs under control (non-exposed) conditions, it was observed that that C3-1, C3-3, and CD109 showed significant higher levels of expression in the resistant (BS90) when compared to susceptible snails (NMRI), while C3-2 and TEP-1 had significantly higher expression levels in the susceptible compared to resistant snails (**Figure 4**).

**Figure 5:**
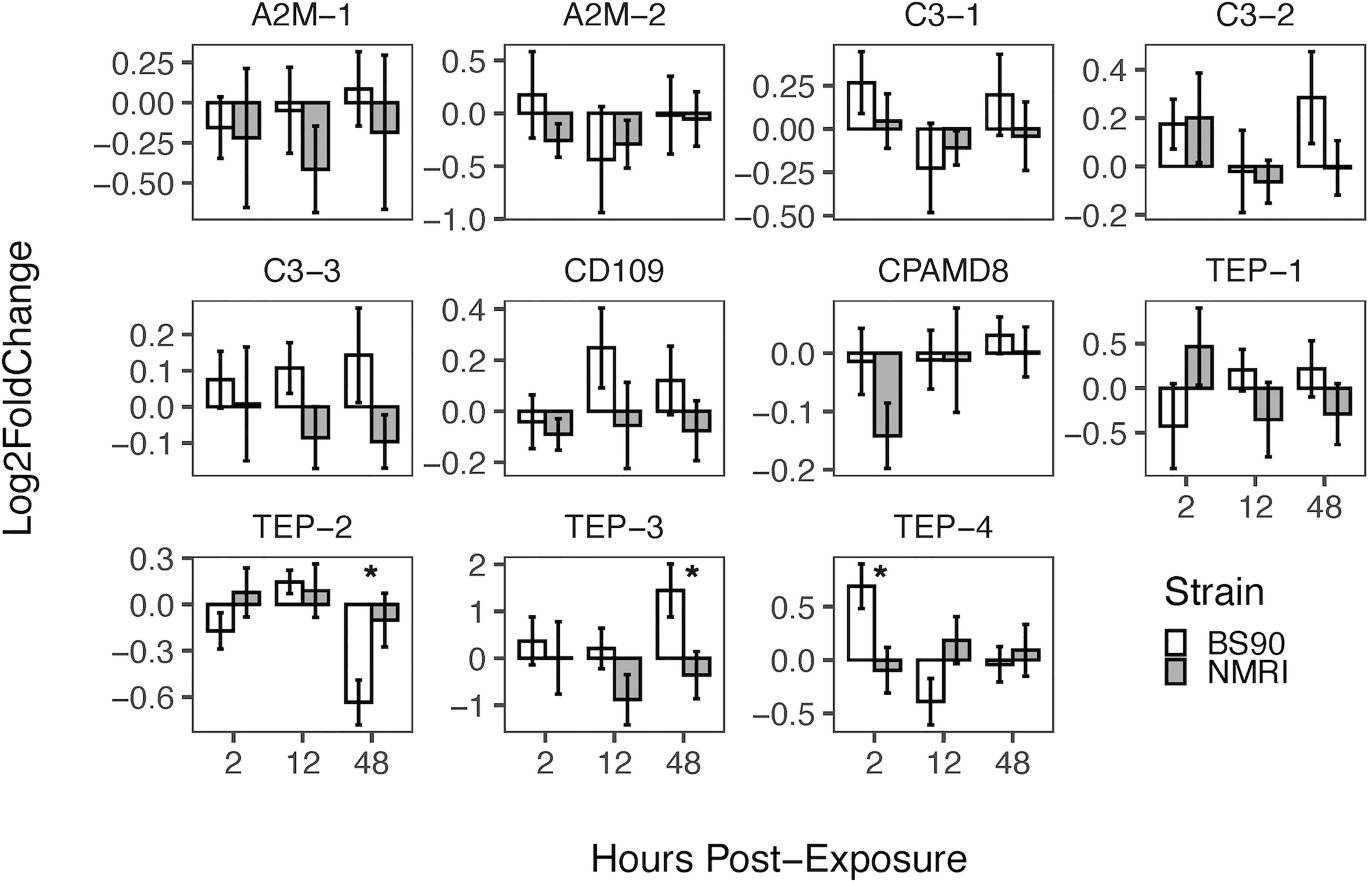
Expression of TEPs in *B. glabrata* snails exposed to *S. mansoni* miracidia. The expression of TEP transcripts was measured *via* qRT-PCR in resistant (BS90, white bars) and susceptible (NMRI, gray bars) at 2, 12 and 48 h after infection with 15 *S. mansoni* miracidia. To compare TEPs gene expression between BS90 and NMRI snail strains post-exposure, log-transformed expression levels of exposed snails were calculated relative to control snails for each strain using the Pfaffl comparative method (n=9). Statistical analysis was performed using the non-parametric Mann-Whitney test due to non-normal distribution of the data. Values were considered significant if p ≤ 0.05.

Furthermore, when comparing the expression of TEPs in snails exposed to *S. mansoni* miracidia, we found that during the early response (2 h), TEP-4 was significantly upregulated in resistant snails (**Figure 5**). At 12 h, there was no significant difference in any of the TEPs expression between strains, while at the 48 h time point TEP-3 was upregulated and TEP-2 was downregulated in the resistant (BS90) compared to susceptible (NMRI) snails (**Figure 5**). Finally, TEP-3, and TEP-4 transcripts were upregulated at a higher level upon exposure to *S. mansoni*, especially TEP-3 in resistant (BS90) snails (**Figure 5**).

## Discussion

### *B. glabrata* have a diverse collection of TEP proteins

The main objectives of this this study were to verify the expression and coding sequences of 11 previously identified members of the TEP superfamily in *B. glabrata* resistant and susceptible strains (10, 56), and to test their modulation upon exposure to *S. mansoni* miracidia. Results confirmed the expression of all 11 *B. glabrata* TEPs in both susceptible (BB02) and resistant (BS90) snail strains and revealed constitutive differences between the two snail strains. Ten of these TEP sequences were described as novel proteins in both BB02 (10) and Bre1 (56) susceptible strains, while TEP-1 has been previously characterized and found to interact with *S. mansoni* tegumental glycoproteins (20, 22, 23). TEP sequences from *B. glabrata* BB02 and BS90 snail strains were manually verified *via* PCR amplification and Sanger sequencing using previously identified transcripts in the VectorBase database (9, 10). These sequences comprised 11 discrete and unique genes, and to the best of our knowledge, the *B. glabrata* TEP superfamily of proteins. Results from the NT sequence analysis using blast and phylogenetic analysis grouped *B. glabrata*’s TEPS into the three traditional TEP subfamily clades. These included three members from the A2M group (A2M-1, A2M-2, CPAMD8), three complement C3-like molecules (C3-1, C3-2, C3-3), and four classical TEP proteins (TEP-1, TEP-2, TEP-3, TEP-4) as well as a CD109 homolog. Based on length similarity with known homologs, identification of putative start and stop codons, and presence of 5’- and 3’-untranslated regions, we determined that seven of the 11 molecules were complete (A2M-1, C3-1, C3-2, CD109, TEP-1, TEP-2, and TEP-4). The four remaining sequences (A2M-2, C3-3, CPAMD8, and TEP-3) were considered incomplete and missing characteristic protein domains necessary for definite characterization. A2M-2 and CPAMD8 were missing approximately 60% of the expected protein sequence, and therefore, categorized according to NCBI’s protein blast predictions alone. Finally, C3-3 and TEP-3 were categorized based on phylogenetic analysis. This characterization was supported by a recent study identifying TEPs in the *B. glabrata* Bre1 snail strain, albeit differences in designation for several molecules compared to the ones presented in this study (56). A phylogenetic tree depicting these naming differences is presented in **Supplemental Figure 1**.

All together, these observations confirmed that the genome of *B. glabrata* codes for multiple TEP genes as seen with many other organisms (25, 29). In addition, the closest homologs for all *B. glabrata* TEPs were identified in other invertebrate clades, specifically mollusks, providing additional information regarding the evolutionary relationship of these genes (25, 64).

### TEP sequences in *B. glabrata* resistant and susceptible snails have small differences

One major objective of this study was to identify differences in TEP sequences between the resistant and susceptible snail strains, as these could have a direct correlation to anti-schistosome immune mechanisms. Known members of the TEP superfamily in other organisms are approximately 1500 AAs, which corresponds with the length of the complete TEPs described in both BB02 and BS90 snails in this study, with varying size from 1387 to1754 AAs. Comparison of TEPs for which we had complete sequences, showed identical lengths between BB02 and BS90 strains, with the exception of TEP-1, which showed 5 AA differences between strains. Although the potential functional consequences of such length divergence remain to be determined, the observed difference suggests that even within the same species, differences exist at the genetic level, and may be due to allele prevalence in these strains.

In terms of sequence identity, BB02 and BS90 TEP NT and protein sequence alignments revealed high similarity, however, differences between the two strains still exist. Comparisons of A2M-1 revealed 27 NT differences which all translated to AA differences. In contrast, C3-1 and TEP-4 also contained many NT differences (70 and 44, respectively), but only a few AA differences (six and nine respectively). Finally, TEP-1, -2, and -3 showed to be the most divergent TEPs, with over 60% of NT differences (75%, 61%, and 78%, respectively) and translating into 47, 45, and 42 AA changes respectively. The AA mismatches between strains for TEP-1, -2, and -3 were found primarily around and within the A2M domain, which is known to be a hypervariable region in TEPs (31, 65). The polymorphisms within *B. glabrata* TEP genes could be another variable associated with the different levels of susceptibility observed in wild populations of snails. For example, in *A. gambiae* mosquitos, the presence and expression of different AgTEP-1 forms was associated with alleles correlating with resistance or susceptibility to *Plasmodium* infections (66, 67).

To further characterize differences in TEPs between BB02 and BS90 strains, corresponding sequences were aligned and the characteristic TEP superfamily protein domains (A2M_N, A2M_N_2, A2M, A2M_comp, and A2M_recep) compared and analyzed (**Figure 2**). Putative protein sequences from BB02 and BS90 snails contained those same conserved domains with similar organization as TEPs previously characterized in other organisms such as humans, flies, and mosquitoes (31, 68).

Three of the four incomplete sequences, A2M-2, CPAMD8, and C3-3, were missing domains that prevented their final characterization. Representative domains in CPAMD8 molecules include the Methyltransf_FA (farnesoic acid 0-methyl transferase) domain, which in insects is involved in the biosynthesis of juvenile hormone (69), and the kazal domain, which functions as a serine protease inhibitor (51, 70). Absent also in the snail sequences, is the A2M_2 domain found in CPAMD8 homologs. Instead, the snail’s CPAMD8 contains the complement_C3_C4_C5 domain in this same region, which is characteristic of the C3-like molecules. This lack of definition is reflected in the phylogenetic analysis, where A2M-2 and CPAMD8 were assigned to a separate group due to their incomplete status (**Figure 1**). Furthermore, the alignment of BB02, BS90, and Bre1TEP sequences revealed that both A2M-2 and CPAMD8 reported in this study were instead characterized as macroglobulin complement-related proteins (MCR) by Duval etal. (56). MCRs are TEP proteins containing a low-density lipoprotein receptor (LDL) domain localized at the same region that complement molecules have the anaphylatoxin (ANATO) domain, or where A2M’s have the bait region (65, 71, 72). The similarity between *B. glabrata* TEP sequences suggests the likelihood that these two incomplete molecules could be MCR homologs. However, as the A2M-2 and CPAMD8 transcripts from BB02 and BS90 snails are not yet completely verified, these two TEPs were characterized following the alignments to closest homologs from NCBI.

Molecules in the complement C3-like subgroup have a unique ANATO domain which contains characteristic cleavage points. Traditionally, C3 is secreted as a full-length precursor that once cleaved is activated producing C3a and C3b fragments. The C3a fragment, or anaphylatoxin, is involved in chemotaxis, recruitment of immune cells, and induction of other immune responses such as inflammation; while the C3b fragment exposed thioester is involved in opsonization and lysis by binding to cells and other targets (65, 68). The ANATO domain was not identified by the software tools used in this study in any of the C3-like TEPs from *B. glabrata*. However, *B. glabrata*’s activating enzymes may recognize different cleavage sites in the ANATO domain that have yet to be characterized. The C3-3 sequence remains incomplete and is currently missing the NTR_complement_C345C and C345C/NTR domains which are important for complement activation and the formation of the membrane attack complex (73, 74), preventing its definite assignment as a complement-like molecule.

Although many of the characteristic domains were identified and supported the idea that *B. glabrata* has a diverse TEP family, further studies are needed to characterize these TEPs functionally.

### TEPs in susceptible and resistant snails showed amino acid substitutions

Although AA mismatches between resistant (BS90) and susceptible (BB02) strains could be of importance, the apparent lack of pattern(s) did not allow to draw any conclusions relative to functionality. Further studies are needed to assess if these changes alter protein structure/function. The possibility that these mismatches represent different alleles in susceptible and resistant populations and their relationship with *Schistosoma* parasites is worth exploring as demonstrated previously in mosquitoes (66, 67). When comparing BB02 to BS90 sequences, A2M-1, TEP-1, -2 and -3 contained an array of AA mismatches near and within the A2M domain, a known hypervariable region in other species homologs (65, 71, 72). Interestingly, although BB02 and BS90 snails are members of the same species, their TEP sequences contained many AA mismatches in this hypervariable region. Except for TEP-4, which had higher sequence conservation in this region. The diversity among snail TEPs of different strains suggests that these molecules may have versatile functional roles and respond to different targets. Alternatively, these AA substitutions may correlate to differences in target binding affinity, leading to various levels of immune efficacy in snail strains. That is, snails with specific alleles could exhibit a resistant phenotype when confronted by a particular immune attack, while presence of other alleles may render the hosts susceptible. In *Drosophila*, the hypervariable region of TEP2 was shown to undergo alternative splicing (31), and data from Duval etal. (56) suggested this may be the case for one or more of the *B. glabrata* TEPs. Lastly, the diversity seen in the classical TEP subgroup suggests that during an immune response the presence of certain TEPs can also have negative effects, as shown in the study by Shokal etal. (54), where inactivation of TEPs 2 and 6 offered a protective effect when *Drosophila* flies were infected with bacteria. It is unlikely that all AA mismatches found between the *B. glabrata* BB02 and BS90 strains are biologically relevant and cause structural differences altering function and/or specificity, but this remains to be investigated.

### Thioester region analysis of *B. glabrata* TEPs suggest classical biological function

When comparing BB02 to BS90 TEPs, nine of the eleven sequences contained the same TER sequence. Six of these nine, A2M-1, C3-1, TEP-1, TEP-2, TEP-4, and CD109, had the representative (G)CGEQ AA sequence in both snail strains, suggesting these TEPs likely have similar immunological function as those described in other organisms (25, 31, 37, 54, 66, 72, 75, 76). However, a TER could not be identified in C3-3 and CPAMD8, regardless of strain. CPAMD8 is incomplete and it would be premature to make any conclusions regarding TER integrity in this sequence. In contrast, C3-3 was found to have a truncated form of the complement_C3_C4_C5 domain, and it is possible that the TER, normally located in this domain, was modified or lost and thus its homology to complement component C5 (44, 45).

Three TEPs (A2M-2, C3-2, and TEP-3) had recognizable but alternative TER sequences in both BB02 and BS90 strains (**Figure 3**). A2M-2 contained mutated AA sequences in TER positions two and five, resulting in the motif GGGEM. These two mutations locate to the most important AA positions forming the internal thioester and consequent binding to targets. Theoretically, the R-groups from glycine and methionine found in the alternative sequence, can form weak hydrophobic interactions for protein structure and function to be retained. C3-2 contained mutated AAs in TER’s positions two, three, and five, coding for GLMEE, which would also affect the formation of the internal thioester. In this specific sequence, the R-groups in those AAs would not associate, as leucine is hydrophobic and glutamate is negatively charged. In conclusion, it appears that A2M-2 and C3-2 possess nonfunctional TERs as their mutations would prevent the formation of the classic thioester bond. However, it is possible that the structural integrity and functionality of these two molecules may not be affected, as seen for example with C5 and MCR molecules which lack the characteristic TER but still maintain their functions (65, 68, 77, 78).

TEP-3 contained one mutated AA in the TER at position two, resulting in GSGEQ. This mutation occurs in one of the most important positions in the formation of the internal thioester. Interestingly, this mutation replaced the characteristic cysteine residue with a serine. Cysteine and serine are similar in size, and both AAs have polar, uncharged, nucleophilic R-groups, with cysteine containing a sulfhydryl group whereas serine has a hydroxyl group. This suggests that TEP-3 may retain normal conformation but not have a functional TER due to the inability to form a thioester bond. Since thioester groups are more reactive than ester functional groups, it is still unknown whether TEP-3’s TER remains functional. However, this TER conformation is not unique to *B. glabrata*, as an MCR molecule in *D. melanogaster*, also referred as TEP6, was also found to contain a serine residue in place of the cysteine (72, 78). Interestingly, this MCR/TEP6 molecule was responsible for the phagocytosis of *Candida albicans* (72), suggesting that alternative TER domains maintain immune function. This was later supported in a study where several TEPs (TEP2, TEP4, and TEP6) in *D. melanogaster* were found to have different functions depending on the presence and structural characteristics of the TER domain (77).

In summary, findings from the comparative analysis of *B. glabrata* BB02 and BS90 TER, suggest that the differences among TEP members and snail strains could be what allows immune flexibility and capacity to recognize a variety of pathogens. Furthermore, alternative TERs in combination with small number of AA differences in hypervariable regions may aid in diversification and refine pathogen detection levels, and may in part explain differences in snail susceptibility and resistance to schistosome parasites.

### TEPs response to schistosome infection differs between resistant and susceptible snails

Understanding and characterizing the cellular and molecular mechanisms associated with snail defense responses to *S. mansoni* remains a relevant topic of study. In resistant snail strains, recognition of the parasite triggers an initial cellular response by hemocytes, which migrate to the infection site and eventually kill the parasites during encapsulation and cytotoxic reactions (reviewed in 8). Several snail immune responses have been characterized, notably the hemocytes’ production of reactive oxygen and nitrogen species (14, 15), which are directly involved in parasite killing. Other cellular and humoral responses, include the presence of the macrophage migration-inhibitory factor (MIF), various fibrinogen-related proteins (FREPs) (79), biomphalysin (80), and TEPs, all thought to be involved in recognition and opsonization of pathogens (reviewed in 24, 81).

Here, we focused on the TEPs response in the initial stages of infection by *S. mansoni* miracidia. When comparing TEP expression between resistant (BS90) and susceptible (NMRI) snails, we found that C3-1, C3-3 and CD-109 showed a higher constitutive expression in resistant snails compared to their susceptible counterparts (**Figure 4**). In contrast, C3-2 and TEP-1 showed a significant higher level of constitutive expression in susceptible snails compared to resistant snails (**Figure 4**). Interestingly, those TEPs with higher constitutive expression in the resistant strain (C3-1, C3-3, and CD-109) show no further increase upon exposure to parasites, suggesting they may play a role as sentinel molecules, and be key to triggering a fast and/or more efficient/specific response to *S. mansoni*. Higher constitutive expression of specific genes in *B. glabrata* has been previously reported in association with resistant phenotypes (58, 79, 82). Differences in the constitutive expression of the enzyme Cu/Zn superoxide dismutase, a component of the ROS cascade, was found to be higher in hemocytes of resistant snails compared to susceptible (83). Similarly, Larson etal. (84), found that resistant snails had higher number of hemocytes, and/or these cells expressed higher levels of several immune-related genes including the potential hemocyte chemoattractant, allograph inflammatory factor (AIF). In this study, C3-2 and TEP-1 showed a significant higher level of expression in susceptible compared to resistant snails. This observation that snail strains have differential protein profiles has been expanded in a recent study looking at single-cell RNA-seq in hemocytes of both susceptible and resistant *B. glabrata* snails where they found that based on their gene expression profile, hemocytes from resistant snails appear to be better prepared for immune challenge that their resistant counterparts (24).

In *S. mansoni* exposure experiments, only resistant snails (BS90) showed significant increase in TEP expression, specifically TEP-4 early after infection (2 h), and later with TEP-3 at 48 h, suggesting that these molecules may play a central role in resistance to this parasite (**Figure 5**). Interestingly, TEP-4 was also reported to be expressed in hemocytes in a previous study (TEP-2 in 56), and be present in plasma proteins that bind to the surface of sporocysts (20). TEP-4 is also the molecule that was found to have multiple alternative splicing variants (56), but in depth analysis was not performed to differentiate between them in this study. In contrast to earlier studies showing that TEP-1 is increasingly upregulated in resistant snails exposed to *S. mansoni* miracidia (23), we only observed a slight upregulation of TEP-1 transcript in susceptible snails. The discrepancy in results between these studies could be due to the different strains of snails and parasites used and the consequent differences in compatibility between them. Alternatively, the fact that our results showed little increase in TEP-1 expression could also be due to the tissues tested; Portet etal. (23), used whole snails to test TEP-1 response to immune challenge, while only the headfoot region was used in the present study. Previously, it was reported that TEPs are not expressed evenly in all snail tissues (56), and in this study, the headfoot region of the snails was selected as it is the tissue where miracidia penetrate and reside during the initial stages of infection. Several other TEPs are upregulated in response to parasite exposure including C3-3 and CD109, but the increase in expression was not found to be significant; nonetheless, the expression pattern is different between strains (**Figure 5**).

Results from the current study showed that none of the TEPs had significant upregulation in susceptible snails. One possible explanation is that the susceptible snails are unable to recognize the invading parasite due to the absence or insufficient levels of specific constitutively expressed molecules; or perhaps, the inability of susceptible snails to destroy the parasites is due to specific alleles (susceptible) responding when compared to those found expressed in resistant strains. Indeed, Bender etal. (85) showed an association between a specific SOD1 allele (a component of the oxidative burst) and snail resistance to *S. mansoni*. As well, Li etal. (79) found that only resistant snails expressed FREP2, which in turn increased the killing activity of hemocytes against *S. mansoni* sporocysts. Furthermore, allelic variations in several genes within the Guadeloupe resistance complex (GRC) in *B. glabrata* have also been found to correlate with schistosome-resistance/susceptibility phenotypes (86, 87). Alternatively, the apparent lack of efficient immune response could be due to blockage or inactivation by products released/produced by transforming miracidia (LTPs) (20). *Biomphalaria*’s response to schistosome infection is known to involve a multitude of cellular and humoral immune players, responding to the varied antigenic cues from the parasites themselves (88–90). The diversity of TEPs’ alleles found in different snail populations may reflect adaptations to the variability of indigenous PAMPs.

Further studies would be needed to directly link specific TEP alleles to snail phenotypes. As mentioned earlier, so far, only TEP-1 was shown to be recruited to the tegumental surface of the parasite by FREP-3 and potentially serve as an opsonin, while further recruiting other humoral factors such as biomphalysin and FREP-2 (22, 23, 79). The fact that *B. glabrata* expresses a diversity of TEPs, suggests that there may be specificity on their expression depending on the immune challenge and the tissue(s) affected; Portet etal. (23) reported that TEP-1 was differentially expressed in snail tissues, with particular high constitutive expression levels in hemocytes, ovotestis and the headfoot region, and that it was upregulated in response to *Micrococcus luteus* in addition to *S. mansoni*.

This specific and discriminatory role was proposed for TEP-1 in *B. glabrata* (56), and is supported by previous studies in arthropods, where unique TEPs are involved in clearing specific pathogens (33, 54, 77, 91–96). The susceptibility trait of snails to schistosomes may not necessarily correlate to susceptibility to other pathogens, and it would be interesting to compare TEPs responses to various other pathogens or immune stressors.

### Concluding remarks

This study reports the characterization of 11 members of the TEP superfamily and their differences in susceptible (BB02) and resistant (BS90) *B. glabrata* snails. TEP homology through phylogenetic analysis, sequence length, and domain architecture of assembled transcripts confirm that *B. glabrata* TEP sequences are very similar to characterized homologs in vertebrates and invertebrates. The comparative analysis conducted in this study between BB02 and BS90 AA TEP sequences demonstrated that *B. glabrata* strains have minor structural and thus possibly functional differences in these molecules, including some in conserved domains such as the thioester region. The presence of the traditional TER in many of the *B. glabrata* TEPs suggests that these molecules have similar functions as those described in other organisms. This diversity of TEP molecules and their expression in *B. glabrata* illustrates another example of the broad range of protective components invertebrates have at their disposal.

Immune system components, like the TEPs, may be an important part in the functional mechanisms behind snail susceptibility and resistance. Furthermore, differential expression of particular TEP components in resistant and susceptible snail strains in response to *S. mansoni* parasites, suggests that the ability of snails to produce a particular protein may not be sufficient to offer protection, and instead, the constitutive level of certain molecules may give an advantage to individual snails (24, 82). Alternatively, effective recognition and successful killing of invading miracidia may require a very specific and consecutive order of events (such as the expression of a particular TEP), that if not accomplished at the proper time and concentration, could result in the animal succumbing to infection. In this scenario, the interaction of parasite products with specific TEPs may further hinder defense mechanisms by modulating host immunity.

This study on *B. glabrata* TEPs expands our current knowledge on the diversity of this family of proteins in molluscan species and related invertebrate organisms. Finally, these findings offer the basis to continue the detailed study of *B. glabrata’s* TEPs structure, expression and localization, to further characterize their potential role in the schistosome-resistance traits of snail populations.

## Data availability statement

The datasets presented in this study can be found in online repositories. The names of the repository/repositories and accession number(s) can be found in the article/**Supplementary Material**.

## Ethics statement

The animal study was reviewed and approved by American Association for Accreditation of Laboratory Animal Care and the University of Wisconsin-Madison Institutional Animal Care and Use Committee (IACUC, Animal Welfare Assurance No. A3368-01).

## Author contributions

MJ wrote most of the portions of the first draft of the manuscript; MJ and DN performed experiments, collected and analyzed data, prepared figures, and tables, and wrote the manuscript; GA and KA performed experiments, collected and analyzed data; JN performed the statistical analysis, wrote a section fo the manuscript and prepared figures; DN, YT, and CM contributed to the design of the study; YT and CM laboratory facilities were used to carry out for experiments; CM wrote portions of the manuscript. All authors contributed to the manuscript revision and approved the submitted version.

## Funding

This research was funded by the Department of Pathobiological Sciences, School of Veterinary Medicine at the University of Wisconsin and by the NIH grant 1-SC2-AI-133645 to Maria G. Castillo at New Mexico State University.

## Conflict of interest

The authors declare that the research was conducted in the absence of any commercial or financial relationships that could be construed as a potential conflict of interest.

## Publisher’s note

All claims expressed in this article are solely those of the authors and do not necessarily represent those of their affiliated organizations, or those of the publisher, the editors and the reviewers. Any product that may be evaluated in this article, or claim that may be made by its manufacturer, is not guaranteed or endorsed by the publisher.
