# Supplementary material for: Molecular characterization of thioester-containing proteins in Biomphalaria glabrata and their differential gene expression upon Schistosoma mansoni exposure
Source: Front Immunol. 2022 Jul 27;13:903158. doi: 10.3389/fimmu.2022.903158 (PMC9363628; doi:10.3389/fimmu.2022.903158)
Supplement: Supplementary file 1 [file DataSheet_1.docx]

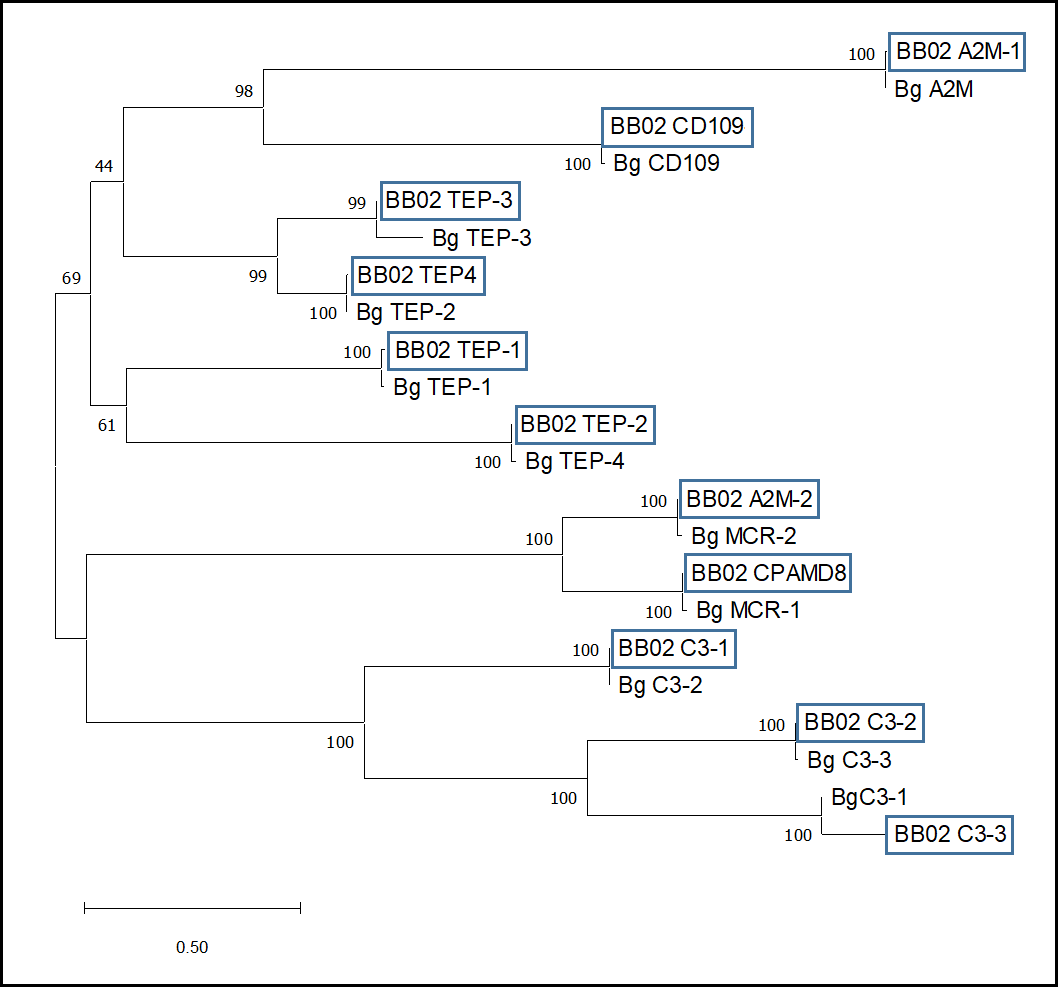


**Supplemental Figure 1. Phylogenetic Tree Comparing *Biomphalaria* *glabrata* BB02 and Bre1 TEP sequences.** MUSCLE was used to align TEP nucleotide sequences from the *B. glabrata* BB02 strain from this study against those from the Bre1 strain (Portet *et al*., 2018; Duval *et al*., 2020). Following alignment, a phylogenetic analysis using MEGA-X (Kumar *et al*., 2018) was used to construct a Neighbor-Joining tree (Saitou & Nei, 1987) with the James-Thornton-Taylor model (Jones *et al*., 1992) and a Bootstrap value of 500 replications. BB02 TEP sequences are labeled with a blue box.
