# Supplementary material for: Molecular characterization of thioester-containing proteins in Biomphalaria glabrata and their differential gene expression upon Schistosoma mansoni exposure
Source: Front Immunol. 2022 Jul 27;13:903158. doi: 10.3389/fimmu.2022.903158 (PMC9363628; doi:10.3389/fimmu.2022.903158)
Supplement: Supplementary file 2 [file Table_1.docx]

**Supplemental Table 1. PCR Primers**. PCR primers that were used to amplify segments of each individual *B. glabrata* TEP sequence. PCR verified indicates that the PCR product was visible in agarose gel electrophoresis and the expected band size was observed.

| **TEP Primer** | **Sequence 5' - 3'** | **T_m_ (^o^C)** | **T_a_ (^o^C)** | **Product Length** | **PCR Verified** | **Old Characterization** |
| --- | --- | --- | --- | --- | --- | --- |
| A2M-1 F1 250 | GCTTACCTCTCGCTAACTCAAT | 60.8 | 54 | 250 | Yes | N/A |
| A2M-1 R1 250 | AGGCGTGTCTTGTACATTTCT | 58.7 |  |  |  |  |
| A2M-1 F2 738 | CGAGTAGAAGCCTCACACTATTT | 61 | 55 | 738 | Yes | N/A |
| A2M-1 R2 738 | GAGCGCATACTTTGCCTTTG | 60.4 |  |  |  |  |
| A2M-1 F3 806 | AGCCAGGTCAGAAAGTGATG | 60.4 | 55 | 806 | Yes | N/A |
| A2M-1 R3 806 | ACACAGGCTGAAGAGGAATG | 60.4 |  |  |  |  |
| A2M-1 F4 804 | AGGCACTGGAGTTGTTGTTAAT | 58.9 | 55 | 804 | Yes | N/A |
| A2M-1 R4 804 | CCATCAGGGAGGATGTGATAGA | 62.7 |  |  |  |  |
| A2M-1 F5 788 | CTGGTCAAGTCTGGTCACATAA | 60.8 | 55 | 788 | Yes | N/A |
| A2M-1 R5 788 | CTCATACTGGCAGGTACCATAAA | 61 |  |  |  |  |
| A2M-1 F6 674 | CTACTGGGAGGAAACCATCAAG | 62.7 | 55 | 674 | Yes | N/A |
| A2M-1 R6 674 | ACTGGAGGGACAGGAAGAA | 60.2 |  |  |  |  |
| A2M-1 F7 987 | GTGCATCCAGAAACAGGATTTG | 60.8 | 55 | 987 | Yes | N/A |
| A2M-1 R7 987 | TATAGGAGCCCAAGACTCTACC | 62.7 |  |  |  |  |
| A2M-1 F8 711 | GCAGTGGAAAGGACTCATAACA | 60.8 | 55 | 711 | Yes | N/A |
| A2M-1 R8 711 | GAGTTCCAGCTCCACAACAATA | 60.8 |  |  |  |  |
| A2M-1 F9 306 | GACCCTGAATTTGGACCTGAA | 60.6 | 55 | 306 | Yes | N/A |
| A2M-1 R9 306 | TGTGTTTCTGGTGGTCTTGG | 60.4 |  |  |  |  |
| A2M-1 F10 227 | GATGTACCTGTGGTCTGCTTAC | 62.7 | 54 | 227 | Yes | N/A |
| A2M-1 R10 227 | AGTGTTGCTGGACAGTTCAA | 58.4 |  |  |  |  |
| A2M-2 P1 696 F | ACCATGCATGAAGGACAAGATA | 58.9 | 56 | 696 | Yes | N/A |
| A2M-2 P1 696 R | CGCAGTTAGCCAGAGATTAGAG | 62.7 |  |  |  |  |
| A2M-2 P2 978 F | AAGCAGGAATACCGACCTAATC | 60.8 | 56 | 978 | Yes | N/A |
| A2M-2 P2 978 R | CATAGTCATGTCACGCTCTAACT | 61 |  |  |  |  |
| A2M-2 P3 514 F | CAGCAAACAAGGCGGATAAG | 60.4 | 56 | 514 | Yes | N/A |
| A2M-2 P3 514 R | GAGGTCAAGCTACGTTCAGTAT | 60.8 |  |  |  |  |
| A2M-2 P4 283 F | GCTTCAGCTTACTGTCACATC | 60.6 | 56 | 283 | Yes | N/A |
| A2M-2 P4 283 R | AACCCGAGACTGCACATAG | 60.2 |  |  |  |  |
| CPAMD8-1 P1 806 F | TGTTCAGCATTTCAGCGTATTT | 57.1 | 54 | 806 | Yes | N/A |
| CPAMD8-1 P1 806 R | AGAGCTTCCATTGCCAGTATAG | 60.8 |  |  |  |  |
| CPAMD8-1 P2 850 F | CCATGCACATCCTATTCCCTTA | 60.8 | 54 | 850 | Yes | N/A |
| CPAMD8-1 P2 850 R | AGAGTCGTTTCTGCCACTAAAT | 58.9 |  |  |  |  |
| CPAMD8-1 P3 542 F | TGACCCAAATGAAGAGCCTATAA | 59.2 | 54 | 542 | Yes | N/A |
| CPAMD8-1 P3 542 R | AGTAGGTAGCAGCCATCAATAAA | 59.2 |  |  |  |  |
| CPAMD8-1 P4 586 F | GCTCCTACCAATGTCCCTATTG | 62.7 | 56 | 586 | Yes | N/A |
| CPAMD8-1 P4 586 R | CCTTTGTGTGGCCTCAATCTA | 60.6 |  |  |  |  |
| CPAMD8-1 P5 518 F | GTGAGCTGATGGAAATACCTTAAT | 59.4 | 54 | 518 | Yes | N/A |
| CPAMD8-1 P5 518 R | GCATGATATGAAAGGATGCATTTG | 59.4 |  |  |  |  |
| C3-1 F1 481 | ACGGATTACCCGGAGAGGAA | 62.4 | 57.4 | 481 | Yes | N/A |
| C3-1 R1 481 | TGACAGCGCTAGTTGTCCTG | 62.4 |  |  |  |  |
| C3-1 F2 831 | CAGGACAACTAGCGCTGTCA | 62.4 | 57.4 | 831 | Yes | N/A |
| C3-1 R2 831 | TCTCCGACTGCAGGAGTGTA | 62.4 |  |  |  |  |
| C3-1 F3 741 | ATGCCAGGCTGACTGATACG | 62.4 | 57.4 | 741 | Yes | N/A |
| C3-1 R3 741 | TGAGTTCTTGGGTGGCGAAG | 62.4 |  |  |  |  |
| C3-1 F4 883 | CTGCCGATGACCCCTAAGAC | 64.5 | 57.4 | 883 | Yes | N/A |
| C3-1 R4 883 | CACTTGGGTCCAGCATGACA | 62.4 |  |  |  |  |
| C3-1 F5 920 | CTGTCATGCTGGACCCAAGT | 62.4 | 57.4 | 920 | Yes | N/A |
| C3-1 R5 920 | GGGCTGTTTGGTGTACCAGT | 62.4 |  |  |  |  |
| C3-1 F6 760 | AGGAGGATTACGAGGGGCAT | 62.4 | 57.4 | 760 | Yes | N/A |
| C3-1 R6 760 | GGTCTGACAGATCGTCGCTT | 62.4 |  |  |  |  |
| C3-1 F7 883 | CGTACAGCGCCTCAGTCATC | 64.5 | 57.4 | 883 | Yes | N/A |
| C3-1 R7 883 | TTTGAGGCGCTTCACAGTCC | 62.4 |  |  |  |  |
| C3-1 F8 723 | AAGAGGTCTGAGCAGGGAAC | 62.4 | 55 | 723 | Yes | N/A |
| C3-1 R8 723 | AACATGGATGTAGGCCAGAAGA | 60.8 |  |  |  |  |
| C3-2 F1 538 | GCGACGTATTGCCAGGATTT | 60.4 | 55 | 538 | Yes | N/A |
| C3-2 R1 538 | CCAGAAGACTGACGGAATGTTAG | 62.8 |  |  |  |  |
| C3-2 F2 584 | AACATTCCGTCAGTCTTCTGG | 60.6 | 55 | 584 | Yes | N/A |
| C3-2 R2 584 | GGTTACTTGTGTACATCAGCTTTG | 61.2 |  |  |  |  |
| C3-2 F3 700 | GGCCAAGTGATCTACTCCATAAA | 61 | 55 | 700 | Yes | N/A |
| C3-2 R3 700 | GCTTCCTGTCCCTTGGAATAA | 60.6 |  |  |  |  |
| C3-2 F4 710 | TCTCTCTACCTGACTCCATCAC | 62.7 | 57.4 | 710 | Yes | N/A |
| C3-2 R4 710 | CTCCTCCATCAGTCCCTTAGA | 62.6 |  |  |  |  |
| C3-2 F5 596 | GATCCTGGAGCCAAACATACA | 60.6 | 55 | 596 | Yes | N/A |
| C3-2 R5 596 | TTCTTCCTGCCTGTCCAATC | 60.4 |  |  |  |  |
| C3-2 F6 718 | GGAAGACCATCTTACCCTTCAG | 62.7 | 55 | 718 | Yes | N/A |
| C3-2 R6 718 | GGTTGAGTCCAGGGCTATTT | 60.4 |  |  |  |  |
| C3-2 F7 800 | GCCTATGGTCTGCTGGTATTC | 62.6 | 57.4 | 800 | Yes | N/A |
| C3-2 R7 800 | CCATGAGGCTTAGGCTTTCTAC | 62.7 |  |  |  |  |
| C3-2 F8 575 | CCATCCACCAGAAGACAAAGA | 60.6 | 55 | 575 | Yes | N/A |
| C3-2 R8 575 | GAGACACAGCCAACTTGAAATG | 60.8 |  |  |  |  |
| C3-2 F9 520 | GGTCTGTCCCAAGGTGATAAAT | 60.8 | 55 | 520 | No | N/A |
| C3-2 R9 520 | CGCTGTTCAGATCCTCGTAAA | 60.6 |  |  |  |  |
| C3-3 P1 546 F | TATGCTAGGGCTGGATGAGA | 60.4 | 55 | 546 | Yes | TEP-5 P1 546 (was TEP-4) (Found to be part of current C3-3) |
| C3-3 P1 546 R | CCCAGACGCATTTCAACTTTAC | 60.8 |  |  |  |  |
| C3-3 P2 637 F | CAAAGACACTCCACCAGGAATA | 60.8 | 58 | 637 | Yes | TEP-5 P2 637 (was TEP-4) (Found to be part of current C3-3) |
| C3-3 P2 637 R | GGACAAGGACTTACCTCTCAAC | 62.7 |  |  |  |  |
| C3-3 F1 423 | TACCATTGAACGCAGACAGAG | 60.6 | 54 | 423 | Yes | N/A |
| C3-3 R1 423 | ACAGCTCTTCCACACAGTATTT | 58.9 |  |  |  |  |
| C3-3 F2 683 | GCATCTCCCAGCATGAGAATAG | 62.7 | 55 | 683 | Yes | N/A |
| C3-3 R2 683 | GGCACATGAACATCAGCAAAG | 60.6 |  |  |  |  |
| C3-3 F3 1868 | TTGAGAGGAGGCAGCAATATG | 60.6 | 55 | 1868 | No | N/A |
| C3-3 R3 1868 | GTACAGGATGGAACTGGATGAC | 62.7 |  |  |  |  |
| C3-3 F4 447 | GTCGTGCCAGAAGTAACCTAAG | 62.7 | 57.4 | 447 | Yes | N/A |
| C3-3 R4 447 | CTCCTCTGTTGATGTGTGAGAAG | 62.8 |  |  |  |  |
| C3-3 F5 344 | TGATGAAGAGGTTGAGAGGAAAG | 61 | 55 | 344 | Yes | N/A |
| C3-3 R5 344 | GGCTACCAGTCTTGTTGAGAG | 62.6 |  |  |  |  |
| TEP-1 685 F P1 | CCACGTAATGTTGTGCCTGG | 62.4 | 57.4 | 685 | Yes | N-TEP F1 |
| TEP-1 685 R P1 | GCTGGCCGAACGTGTATTTG | 62.4 |  |  |  |  |
| TEP-1 730 F P2 | TTGAGTGGAGTCAGCGGAAC | 62.4 | 57.4 | 730 | Yes | N-TEP F2 |
| TEP-1 730 R P2 | GCAGGCTCATGTTCTGTCCA | 62.4 |  |  |  |  |
| TEP-1 557 F P3 | AAGCTAAATGGCAGCTCGGT | 60.4 | 57.4 | 557 | Yes | N-TEP F3 |
| TEP-1 557 R P3 | CTCTGACCGTTCAGCTCCAA | 62.4 |  |  |  |  |
| TEP-1 552 F P4 | CACTACCTGGACAGAACATGAG | 62.7 | 57.4 | 552 | Yes | N-TEP F4 |
| TEP-1 552 R P4 | CCACAGCCAGGACATAGATTT | 60.6 |  |  |  |  |
| TEP-1 619 F P5 | GACGAGACTCCAATGAAGTTGTA | 61 | 57.4 | 619 | Yes | N-TEP F5 |
| TEP-1 619 R P5 | GAAGTGATGGTGTCTGGTACTG | 62.7 |  |  |  |  |
| TEP-1 739 F P6 | GCTCTTTCCTACAGTGGAAGTT | 60.8 | 57.4 | 739 | Yes | N-TEP F6 |
| TEP-1 739 R P6 | CTCCTTCATGTCTGACGAGAATC | 62.8 |  |  |  |  |
| TEP-1 502 F P7 | CTCGCTCAGTCACACGGAAT | 62.4 | 57.4 | 502 | Yes | N-TEP F7 |
| TEP-1 502 R P7 | ACCAGGCTTTGTTCACCACA | 60.4 |  |  |  |  |
| TEP-1 576 F P8 | GACATGAAGGAGCTCCGGTT | 62.4 | 57.4 | 576 | Yes | N-TEP F8 |
| TEP-1 576 R P8 | GACCAGCAGTTGTTCCCTGA | 62.4 |  |  |  |  |
| TEP-1 338 F P9 | AGTTCCAGTACTTGGCTAACAG | 60.8 | 55 | 338 | Yes | N-TEP F9 |
| TEP-1 338 R P9 | GCAAGCATCAGCCTTTCTAAAT | 58.9 |  |  |  |  |
| TEP-1 510 F P10 | ACTGCTCAAAGCCAAGGAG | 60.2 | 55 | 510 | Yes | N-TEP F10 |
| TEP-1 510 R P10 | GACTTGCTGCCGTAAGTAGAA | 60.6 |  |  |  |  |
| TEP-1 748 F P11 | ACCTATAGTCCTCTGGGGCG | 64.5 | 57.4 | 748 | Yes | N-TEP F11 |
| TEP-1 748 R P11 | GGCAACAGTTGAGGCAAACA | 60.4 |  |  |  |  |
| TEP-2 F1 559 | ACTTCCCAGCTCAATGCC | 59.9 | 56 | 559 | Yes | TEP-1 |
| TEP-2 R1 559 | GGGTCTTTGACTCCTAGCC | 62.3 |  |  |  |  |
| TEP-2 F2 549 | CTACCTTCATTCAGACAGACAAGG | 62.9 | 57 | 549 | Yes | TEP-1 |
| TEP-2 R2 549 | CCCTTTGTCCATTACTCACTAGC | 62.8 |  |  |  |  |
| TEP-2 F3 367 | TTGCCAATCACAGTAAAGGC | 58.4 | 54 | 367 | Yes | TEP-1 |
| TEP-2 R3 367 | CTTCCAAATAAGCTGTGTAGCC | 60.8 |  |  |  |  |
| TEP-2 F4 463 | GGCTAGTGAGTAATGGACAAAGG | 62.8 | 56 | 463 | Yes | TEP-1 |
| TEP-2 R4 463 | TTGTCTGGCACAGTTCTTATGG | 60.8 |  |  |  |  |
| TEP-2 F5 366 | TTGAGCAAAGAAGAGATGGC | 58.4 | 54 | 366 | Yes | TEP-1 |
| TEP-2 R5 366 | GATTGTTGATCCAACCTTTGGG | 60.8 |  |  |  |  |
| TEP-2 F6 554 | CAGTGAGCATTGAAGTTAACGG | 60.8 | 56 | 554 | Yes | TEP-1 |
| TEP-2 R6 554 | GAGCTCTTCTTGTACCATTGCC | 62.7 |  |  |  |  |
| TEP-2 F7 543 | GCTTGTCAATTGGTGTTGATGG | 60.8 | 56 | 543 | Yes | TEP-1 |
| TEP-2 R7 543 | CCCATGTTGTGATAGTGTCAGG | 62.7 |  |  |  |  |
| TEP-2 F8 584 | CAGGCGATTATGGCATGTGG | 62.4 | 57 | 584 | Yes | TEP-1 |
| TEP-2 R8 584 | CTCTTTGGAAAGGATGACTTGACC | 62.9 |  |  |  |  |
| TEP-2 F9 490 | AGGAGTCCGCCAATCTTACG | 62.4 | 57 | 490 | Yes | TEP-1 |
| TEP-2 R9 490 | TCTGAGCCTGTGACAGTGG | 62.3 |  |  |  |  |
| TEP-2 F10 633 | CCAGGATTGAGTGATGCTATGG | 62.7 | 56 | 633 | Yes | TEP-1 |
| TEP-2 R10 633 | TCCACCCTGCATTTCTTTATGG | 60.8 |  |  |  |  |
| TEP-2 F11 610 | TCCAATTAGATGCTGCACAAGG | 60.8 | 56 | 610 | Yes | TEP-1 |
| TEP-2 R11 610 | GTTGAGATGAAGCCACCATTAGG | 62.8 |  |  |  |  |
| TEP-2 F12 472 | GGAGATGGTCTGAGGTACTGG | 64.5 | 56 | 472 | Yes | TEP-1 |
| TEP-2 R12 472 | TTTCAGCTATGACTGTGCTTGG | 60.8 |  |  |  |  |
| TEP-3 S1A F1 123 | TGAGTGTACTTCCTATTGATAGATGG | 61.4 | 57 | 123 | Yes | TEP-2 |
| TEP-3 S1A R1 123 | GCCTGTTGTCTGTCTGGAATA | 60.6 |  |  |  |  |
| TEP-3 S1A F2 411 | CTGTGCAGGATGTGGAAGTTA | 60.6 | 55 | 411 | Yes | TEP-2 |
| TEP-3 S1A R2 411 | AGCCTCCTTGCTGTTGTATG | 60.4 |  |  |  |  |
| TEP-3 S1A F3 524 | CCTACATGATCATAGCACCTTCC | 62.8 | 57 | 524 | Yes | TEP-2 |
| TEP-3 S1A R3 524 | TGCTTCAATCTTCCAGTCTCC | 60.6 |  |  |  |  |
| TEP-3 S1A F4 929 | TAAAGTTACCAGCTGACCTTCC | 60.8 | 57 | 929 | Yes | TEP-2 |
| TEP-3 S1A R4 929 | GCGTACTCTGATACTGTCTATTGG | 62.9 |  |  |  |  |
| TEP-3 S1A F5 784 | AGGAGACTGGAAGATTGAAGC | 60.6 | 55 | 784 | Yes | TEP-2 |
| TEP-3 S1A R5 784 | AACATCTCCAGCCCTAATAACC | 60.8 |  |  |  |  |
| TEP-3 S1A F6 596 | AACAACCTCCATCACAGAACC | 60.6 | 57 | 596 | Yes | TEP-2 |
| TEP-3 S1A R6 596 | GATAGGCAGCAGAGTTTGGG | 62.4 |  |  |  |  |
| TEP-3 S1A F7 540 | TGAAGTGACGAGTACAACACC | 60.6 | 55 | 540 | Yes | TEP-2 |
| TEP-3 S1A R7 540 | ATAACCGCATCTGTGAGAGC | 60.4 |  |  |  |  |
| TEP-3 S1A F8 556 | GCCTATCTTCTGGCTATTGACC | 62.7 | 57 | 556 | Yes | TEP-2 |
| TEP-3 S1A R8 556 | CTTGCACAACCAAATGTTCCC | 60.6 |  |  |  |  |
| TEP-3 S1A F9 812 | AGATGCGGTTATCATTCAGTCC | 60.8 | 57 | 812 | Yes | TEP-2 |
| TEP-3 S1A R9 812 | CAGTTGGCATCTGTAGAAGGG | 62.6 |  |  |  |  |
| TEP-3 S1A F10 516 | GCTGTTAGGAGACAACTCTTGG | 62.7 | 57 | 516 | Yes | TEP-2 |
| TEP-3 S1A R10 516 | CCATTGAAGGGCCTCTATTAGG | 62.7 |  |  |  |  |
| TEP-3 S1A F11 692 | CTCTTAATCTTCCACTCCCTTCC | 62.8 | 57 | 692 | No | TEP-2 |
| TEP-3 S1A R11 692 | AACATAGACTGGCAGAGTTGG | 60.6 |  |  |  |  |
| TEP-4 P1 793 F | GAAGTGGTGGACTGACCTTTGA | 62.7 | 58 | 793 | Yes | CD109-2 P1 793 |
| TEP-4 P1 793 R | CCATCGGGTTGGGTGACTTTA | 62.6 |  |  |  |  |
| TEP-4 P2 921 F | ACTTCCCAGCATCCAATCCTAAA | 61 | 57 | 921 | Yes | CD109-2 P2 921 |
| TEP-4 P2 921 R | TCGCGTCTGTGAGAACAACTAC | 62.7 |  |  |  |  |
| TEP-4 P3 746 F | CGATGTTGAGCCAGGAGATGAT | 62.7 | 58 | 746 | No | CD109-2 P3 746 |
| TEP-4 P3 746 R | CGCGGGTTACTGAGTATGGTAAA | 62.8 |  |  |  |  |
| TEP-4 P4 463 F | TGCCAATGGGTCAGCTTCTAT | 60.6 | 58 | 463 | Yes | CD109-2 P4 463 |
| TEP-4 P4 463 R | CTCCACCAAGAGTTGTCTCCTAAC | 64.6 |  |  |  |  |
| TEP-4 920 F5 S1 | GGTTCAGAACTGGCCAGAATA | 60.6 | 55 | 920 | Yes | TEP-2 F5 S1 (Found to be part of current TEP-4) |
| TEP-4 920 R5 S1 | GTGTCTTGTGTGGAGGAGAAA | 60.6 |  |  |  |  |
| TEP-4 851 F6 S1 | ATACTGGCACCAACCAGAAC | 60.4 | 55 | 851 | Yes | TEP-2 F6 S1 (Found to be part of current TEP-4) |
| TEP-4 851 R6 S1 | CGCATTCTTTGCAGACATCAC | 60.6 |  |  |  |  |
| TEP-4 P5 842 F S2 | CTCTGGAGTTGGACTGACTTTG | 62.7 | 57 | 842 | Yes | TEP-2 P5 842 S2 (Found to be part of current TEP-4) |
| TEP-4 P5 842 R S2 | CTGATGGGATGCCGATTTCT | 60.4 |  |  |  |  |
| TEP-4 P6 297 F S2 | AGGCTGACATTGAGAGTATCC | 60.6 | 55 | 297 | Yes | TEP-2 P6 297 S2 (Found to be part of current TEP-4) |
| TEP-4 P6 297 R S2 | GAAGCAACACAACTGGAGTTTA | 58.9 |  |  |  |  |
| CD109-1 F1 336 | TGTGCTTCTGACACCATTGTA | 58.7 | 54 | 336 | Yes | N/A |
| CD109-1 R1 336 | GACACTTTGAGTTGCAGGAAAG | 60.8 |  |  |  |  |
| CD109-1 F2 610 | TGGACTTCTACTTCCGCTTTG | 60.6 | 55 | 610 | Yes | N/A |
| CD109-1 R2 610 | CACCACTGTTGACGTGTATCT | 60.6 |  |  |  |  |
| CD109-1 F3 907 | ACGTCAACAGTGGTGGTATTAG | 60.8 | 55 | 907 | Yes | N/A |
| CD109-1 R3 907 | AACTCTAACGTGGGTGTGATG | 60.6 |  |  |  |  |
| CD109-1 F4 652 | GTCGGTGAGTCAGCCATAATAA | 60.8 | 55 | 652 | Yes | N/A |
| CD109-1 R4 652 | CTTTCCCGTCTACTTCCGTATC | 62.7 |  |  |  |  |
| CD109-1 F5 277 | GATACGGAAGTAGACGGGAAAG | 62.7 | 57.4 | 277 | Yes | N/A |
| CD109-1 R5 277 | CCCTCTTGATGGAATAGGGTAAG | 62.8 |  |  |  |  |
| CD109-1 F6 941 | CAGTAGCCAAGGAACCGTTTA | 60.6 | 55 | 941 | Yes | N/A |
| CD109-1 R6 941 | GCCAGTACCTGACTGCATATC | 62.6 |  |  |  |  |
| CD109-1 F7 272 | GTGATGGGCTGACTGTCTATG | 62.6 | 55 | 272 | Yes | N/A |
| CD109-1 R7 272 | CCATGGCACCTGTAGTTCTT | 60.4 |  |  |  |  |
| CD109-1 F8 470 | CCTGGTCAGCAACAAGGATAA | 60.6 | 55 | 470 | Yes | N/A |
| CD109-1 R8 470 | CATCATCTCCAGCGAAGGTATAG | 62.8 |  |  |  |  |
| CD109-1 F9 854 | CCAAAGTGGACGCTCAAGATA | 60.6 | 54 | 854 | No | N/A |
| CD109-1 R9 854 | GGCCAGCAAACAAACAACA | 58 |  |  |  |  |
| CD109-1 F10 217 | ACACAGACACGGCTGTAATG | 60.4 | 55 | 217 | Yes | N/A |
| CD109-1 R10 217 | TGTTAAAGAGGAAAGAAGCTGTAGT | 59.7 |  |  |  |  |
