# Supplementary material for: Molecular characterization of thioester-containing proteins in Biomphalaria glabrata and their differential gene expression upon Schistosoma mansoni exposure
Source: Front Immunol. 2022 Jul 27;13:903158. doi: 10.3389/fimmu.2022.903158 (PMC9363628; doi:10.3389/fimmu.2022.903158)
Supplement: Supplementary file 4 [file Table_3.docx]

**Supplemental Table 3. Primer Sets used for Q5 Polymerase Verification of 3’ ends.** Annealing temperatures were calculated using the NEB T_m_ Calculator. PCR verified indicates that the PCR product was visible in agarose gel electrophoresis and the expected band size was observed.
