# Supplementary material for: Molecular characterization of thioester-containing proteins in Biomphalaria glabrata and their differential gene expression upon Schistosoma mansoni exposure
Source: Front Immunol. 2022 Jul 27;13:903158. doi: 10.3389/fimmu.2022.903158 (PMC9363628; doi:10.3389/fimmu.2022.903158)
Supplement: Supplementary file 5 [file Table_4.docx]

**Supplemental Table 4. 5' and 3' RACE Primers.** Annealing temperatures were selected using the touchdown PCR guidelines, where the gene specific primer T_m_ < 72^o^C was used at annealing temperatures ranging from 60-65^o^C, and the gene specific primer T_m_ > 72^o^C was used at annealing temperatures ranging from 65-68^o^C. The range for expected product length was 900-1500 base pairs. Verified indicates that the RACE product was visible in agarose gel electrophoresis and the expected band size was observed. N = Nested.
