# Supplementary material for: Molecular characterization of thioester-containing proteins in Biomphalaria glabrata and their differential gene expression upon Schistosoma mansoni exposure
Source: Front Immunol. 2022 Jul 27;13:903158. doi: 10.3389/fimmu.2022.903158 (PMC9363628; doi:10.3389/fimmu.2022.903158)
Supplement: Supplementary file 6 [file Table_5.docx]

**Supplemental Table 5. TEP Homologs and GenBank Accession Numbers used for the Phylogenetic Tree construction.**
