# Supplementary material for: Molecular characterization of thioester-containing proteins in Biomphalaria glabrata and their differential gene expression upon Schistosoma mansoni exposure
Source: Front Immunol. 2022 Jul 27;13:903158. doi: 10.3389/fimmu.2022.903158 (PMC9363628; doi:10.3389/fimmu.2022.903158)
Supplement: Supplementary file 7 [file Table_6.docx]

**Supplemental Table 6. qPCR Primers used.** All qPCR reactions were ran at an annealing temperature of 55^o^C with 350 nM primer concentrations, except the RPS19 control gene, which was ran at 150 nM. PCR verified indicates that the PCR product was visible in agarose gel electrophoresis and the expected band size was observed for both BB02 and BS90 snail strains.
